# Factors associated with health service utilisation for common mental disorders: a systematic review

**DOI:** 10.1186/s12888-018-1837-1

**Published:** 2018-08-22

**Authors:** Tessa Roberts, Georgina Miguel Esponda, Dzmitry Krupchanka, Rahul Shidhaye, Vikram Patel, Sujit Rathod

**Affiliations:** 1 Centre for Global Mental Health, Department of Population Health, Faculty of Epidemiology and Population Health, London School of Hygiene and Tropical Medicine, Keppel Street, London, WC1E 7HT UK; 2 Health Service and Population Research Department, Institute of Psychiatry, Psychology and Neuroscience, King’s College London, London, UK; 3 Department of Social Psychiatry, National Institute of Mental Health, Prague, Czech Republic; 4 Institute of Global Health, University of Geneva, Geneva, Switzerland; 5 Centre for Chronic Conditions and Injuries, Public Health Foundation of India, New Delhi, India; 6 Care and Public Health Research Institute, Maastricht University, Maastricht, Netherlands; 7 Department of Global Health and Social Medicine, Harvard Medical School, Boston, USA

**Keywords:** Common mental disorders, Depression, Anxiety, Treatment seeking, Health service utilisation, Andersen behavioural model, Systematic review, Healthcare access, Barriers to care

## Abstract

**Background:**

There is a large treatment gap for common mental disorders (CMD), with wide variation by world region. This review identifies factors associated with formal health service utilisation for CMD in the general adult population, and compares evidence from high-income countries (HIC) with that from low-and-middle-income countries (LMIC).

**Methods:**

We searched MEDLINE, PsycINFO, EMBASE and Scopus in May 2016. Eligibility criteria were: published in English, in peer-reviewed journals; using population-based samples; employing standardised CMD measures; measuring use of formal health services for mental health reasons by people with CMD; testing the association between this outcome and any other factor(s). Risk of bias was assessed using the adapted Mixed Methods Appraisal Tool. We synthesised the results using “best fit framework synthesis”, with reference to the Andersen socio-behavioural model.

**Results:**

Fifty two studies met inclusion criteria. 46 (88%) were from HIC.

*Predisposing factors:* There was evidence linking increased likelihood of service use with female gender; Caucasian ethnicity; higher education levels; and being unmarried; although this was not consistent across all studies.

*Need factors:* There was consistent evidence of an association between service utilisation and self-evaluated health status; duration of symptoms; disability; comorbidity; and panic symptoms. Associations with symptom severity were frequently but less consistently reported.

*Enabling factors:* The evidence did not support an association with income or rural residence. Inconsistent evidence was found for associations between unemployment or having health insurance and use of services.

There was a lack of research from LMIC and on contextual level factors.

**Conclusion:**

In HIC, failure to seek treatment for CMD is associated with less disabling symptoms and lack of perceived need for healthcare, consistent with suggestions that “treatment gap” statistics over-estimate unmet need for care as perceived by the target population. Economic factors and urban/rural residence appear to have little effect on treatment-seeking rates. Strategies to address potential healthcare inequities for men, ethnic minorities, the young and the elderly in HIC require further evaluation. The generalisability of these findings beyond HIC is limited. Future research should examine factors associated with health service utilisation for CMD in LMIC, and the effect of health systems and neighbourhood factors.

**Trial registration:**

PROSPERO registration number: 42016046551.

**Electronic supplementary material:**

The online version of this article (10.1186/s12888-018-1837-1) contains supplementary material, which is available to authorized users.

## Background

Common mental disorders (CMD) comprise depressive disorders and anxiety disorders, according to the World Health Organisation’s definition [1], and are a leading cause of disability worldwide [2, 3]. Depressive disorders include major depressive disorder and dysthymia, while anxiety disorders include generalised anxiety disorder (GAD), panic disorder, phobias, social anxiety disorder, obsessive-compulsive disorder (OCD) and post-traumatic stress disorder (PTSD). More than 300 million people were estimated to suffer from depression in 2015 (4.4% of the global population), with almost as many affected by anxiety disorders, although there is substantial comorbidity between the two [1].

Despite evidence of effective treatments for CMD [4], there is a large “treatment gap” for CMD globally, with only 42–44% of those affected worldwide seeking treatment for these symptoms from any medical or professional service provider, including specialists and non-specialists, in the public or private sectors [5]. This proportion has been shown to be much lower in low- and middle-income countries, with estimates of as little as 5% seeking treatment, even when traditional providers are also included [6–8].

Within the Global Mental Health literature, these statistics have been used to call for the scaling up of mental health services in order to reduce the treatment gap [9–14], on the assumption that meeting clinical criteria for CMD indicates – or acts as a proxy for – a need for treatment.

Access to health services has been conceptualised as the “fit between the patient and the health care system” [15]. Donabedian (1973) defines access as “a group of factors that intervene between capacity to provide services and actual provision or consumption of services” [16]. Identifying those factors that are associated with seeking treatment for CMD can help us to better understand the reasons for the treatment gap, and inform service planning to expand access to care.

The Andersen behavioural model of health service utilisation [17] provides a useful framework to inform analyses of factors that influence health service utilisation. The Andersen model is a sociological model of health service utilisation that has been extensively applied [18, 19]. This model proposes that the use of health services is affected by:

- one’s *predisposition* to seek help from health services when needed (a product of socio-demographic characteristics, attitudes and beliefs);
- one’s *need* for care (both objective measures and subjective perceptions of one’s health needs); and
- the structural or *enabling* factors that facilitate or impede service utilisation (such as financial situation, health insurance and social support).

In later iterations of the model it was recognised that these predisposing, enabling and need factors can operate at both the individual level and the contextual level [20, 21].

A substantial body of evidence exists on the factors that influence health service utilization for health conditions such as HIV treatment and maternal health care [22–24], and more recently, depression [25]. However, the latter review included treatment-seeking by adolescents and by specific sub-groups of the population, and as such its results may not be generablisable to the general adult population. Furthermore, since depressive and anxiety disorders are closely related and frequently co-occur [26, 27], with many individuals experiencing mixed anxiety-depression disorders [28], we believe that it is more appropriate when studying non-clinical populations to consider the larger construct of CMD rather than separating these disorders, as has been argued elsewhere [29–31]. To date, there has been no comprehensive review of the factors associated with health service utilisation for symptoms of CMDs in the general adult population.

The aim of this review is to investigate factors associated with the use of health services for CMD symptoms, in observational, population-based studies.

Specific objectives are:

1. To identify factors associated with health service utilisation for CMD among adults in the general population, and to assess the quality and consistency of evidence supporting an association between each factor and health service utilisation for CMD.
2. To evaluate the evidence for these associations from high-income countries (HIC) compared to that from low- or middle-income countries (LMIC).

## Methods

The protocol for this study was registered with PROSPERO (registration number 42016046551) [32].

Results are presented according to PRISMA (Preferred Reporting Items for Systematic Reviews and Meta-Analyses) guidelines (see Additional file 1).

### Information sources and search strategy

We searched four databases; MEDLINE, PsycINFO, EMBASE and Scopus. We combined two key concepts (CMD and health service utilisation) using keywords and subject headings in the respective databases. Results were retrieved on 5th May 2016. The search strategy can be found in the Additional file 2. We supplemented the database search by hand searching and reference searches. We only included articles published in English.

### Eligibility criteria

Since the population of interest is the general adult population, we included only population-based studies, defined as community-based epidemiological studies that are representative of the adult population. We excluded studies that focused only on specific sub-populations such as veterans, students, or prisoners, whose experiences may not be representative of the wider population and warrant separate reviews.

The primary outcome measure of interest was any contact with formal health services – including private, public, generalist and specialist – for mental health reasons (also referred to as “treatment-seeking”) by adults aged 18 and above with CMD. Reflecting the definition of the treatment gap, we focussed specifically on use of services as a binary outcome – i.e. any versus no use – rather than volume of treatment received or quality of care.

To be eligible for inclusion, the study must have tested the association between treatment seeking and any other factors. We therefore included only quantitative studies, published in peer-reviewed journals, and excluded narrative reviews and commentaries. We included only studies in which the analyses were restricted to those individuals who either met diagnostic criteria or screened positive for CMD using a standardised instrument.

For the purposes of this article, CMD is defined as those ICD-10 (International Classification of Diseases - 10th Revision) disorders measured by the Clinical Interview Schedule - Revised [33] – often considered the gold standard for measuring CMD [34, 35] – namely, depressive disorders, generalised anxiety disorder, panic disorder, phobias, obsessive compulsive disorder, and mixed anxiety-depression disorder.

We excluded papers that measured only intentions to seek help, or perceived barriers to care, since multiple studies have found that these are not closely correlated with behaviour [36–40].

No restrictions were placed on geographic area or date of publication. Table 1 provides full details of the inclusion criteria applied.

**Table 1:** Inclusion and exclusion criteria applied

	Include	Exclude
Participants	- Population-based studies, in which participants are randomly sampled from a sampling frame that can be reasonably expected to include the majority of the adult population - Studies in which CMD is measured and analyses are restricted to those who “screen positive” for CMD.^a^	- Any studies including people aged under 18 (unless these are presented separately in analyses) - Studies with exclusion criteria that would rule out a large proportion of the adult population (e.g. over-55 s only, people of a particular minority ethnic group only, women who have recently given birth) - Studies in which participants do not live in community settings (e.g. prisoners, inpatients, residents of elderly care homes) or are defined by their occupation (e.g. doctors, police officers, students) - Studies in which all participants have used health services for mental health reasons - Studies that combine people with CMD and those with other conditions and do not report results separately in analyses - Ecological level studies in which CMD is not controlled at the individual level (i.e. it’s not possible to tell whether the people using services are the same individuals who have CMD) - Studies that apply overly restrictive exclusion criteria for participants, e.g. focussed solely on individuals with a specific comorbid condition, or restricted to only specific ethnic groups
Design	- Observational - Quantitative or qualitative comparison of treatment-seekers and non-treatment-seekers - Cross-sectional or longitudinal - Articles published in peer-reviewed journals only	- Reviews/commentaries/opinion pieces - Conference abstracts/dissertations/book chapters - Case studies that lack quantitative evaluation
Outcomes	- Studies reporting on the use/non-use (as a binary variable) of formal, face-to-face health services (either specialist or non-specialist, public or private) for mental health reasons - Timeframe in which service use is measured must be clearly defined (e.g. past 12 months)	- Studies reporting on general health care use (i.e. including for reasons other than mental health problems) - Studies examining use of only one specific treatment type (e.g. antidepressant use only, counselling only) - Studies reporting on volume of treatment (i.e. number of visits to a treatment provider), adherence to treatment or quality of treatment - Studies reporting on rates of detection or referral - Studies reporting on theoretical access rather than actual use (e.g. insurance coverage, being registered with a clinic) - Studies reporting on the use of online or telephone-based services - Studies examining the use of informal care (e.g. friends/family/religious support) or complementary/alternative treatments (i.e. those provided outside of the formal health sector) - Studies reporting on willingness or intentions to use services, or recommendations for service use in case of experiencing CMD symptoms, with no measure of actual behaviour - Studies that report participation in screening as the outcome rather than active treatment-seeking or uptake of services post-screening
Correlates	- Any factors that are correlated with the outcome of interest, including (but not limited to): • demographic factors • health status (e.g. severity/disability/comorbid conditions etc.) • distance/transport to services • insurance coverage • interventions • specific symptoms • behavioural/personality factors • neighbourhood characteristics • characteristics of the healthcare provider • health systems factors • stigma/attitudes towards services	- Studies reporting on the magnitude of the treatment gap, without any correlates of treatment-seeking - Studies that report predictors of service type (e.g. generalist vs. specialist, pharmacological vs. psychological) rather than any vs. no use - Studies reporting barriers and facilitators to the use of health services, without examining the association between these barriers and actual treatment-seeking behaviour
Dates	Any year of publication	
Region	Any country or region	

^a^Defined as any of the following: depression, generalised anxiety disorder (GAD), panic disorder, phobias, obsessive compulsive disorder (OCD), or CMD not otherwise specified

### Study selection

The first author completed title and abstract screening for all references retrieved. Subsequently two researchers (GME and DK) independently screened a random sample of 10% of the references, and inter-rater reliability was calculated at 94%. Full texts were retrieved for all studies included after the title/abstract screening. The first author screened all full texts, while the second author (GME) screened a purposive sample of 10%. At both stages, disagreements were resolved through discussion.

We assessed the quality of the relevant evidence extracted from the included studies using the Mixed-Method Appraisal Tool (MMAT) [41], which has been shown to be quick and reliable to apply [42]. Table 2 sets out the criteria used. Extracted evidence for the purposes of this review was rated as poor, fair, good or excellent if 0–1, 2, 3 or 4 of these criteria were met, respectively. These ratings are not intended to reflect the study quality in relation to its own primary aims, but only of the quality of the evidence that related to this review.

**Table 2:** Operationalisation of quality appraisal criteria, based on the Mixed-Method Appraisal Tool (MMAT)

Criterion	Definition	Example
Appropriate sampling strategy	Population-based sample using a sampling frame that can reasonably be assumed to include the majority of the non-institutionalised adult population. (Justification of sample size was not included in this criterion since none of the included studies justified their sample size with reference to the research questions addressed in this review.)	*Meets criterion:* Simple random sample of households chosen from a government list of residential addresses, then one resident aged > = 18 randomly chosen to participate. *Doesn’t meet criterion:* Males and females sampled through separate means (males at compulsory conscription, females at enrolment on the electoral register).
Sample representative of target population	Sample representative of non-institutionalised adult population, with minimal exclusion criteria applied.	*Meets criterion:* All adults eligible in urban area where study was conducted. Sample representative of urban residents with regard to major socio-demographic factors tested. *Doesn’t meet criterion:* Participants excluded due to age, ethnicity, chronicity of symptoms, comorbid conditions etc.
Appropriate measures used	Validated measure of CMD (either screening tool or diagnostic instrument), timeframe for health service utilisation limited and specified.	*Meets criterion:* CIDI, AUDADIS-IV, CIS-R, SPIKE, PHQ-9, GAD-7, DIS, Burnam depression screener 12 month help-seeking from health services for MH reasons *Doesn’t meet criterion:* Self-defined depression/anxiety, prior receipt of diagnosis Lifetime use of health services (due to limited accuracy of recall)
Acceptable response rate	> 60% response rate for cross-sectional studies > 60% response rate and < 30% attrition rate for longitudinal studies	*Meets criterion:* > 60% response rate across all study sites, or across all major groups compared *Doesn’t meet criterion:* < 60% response rate overall, in some study sites, or for one gender

### Data extraction and synthesis

The following data were extracted for all papers that were included in the full text search: study title, authors, publication date and journal; country; study design; population; CMD measure; outcome (i.e. health service utilisation) measure; and factors associated with the outcome (including null associations). An association was regarded as detected when it was associated with the outcome in the most fully-adjusted model presented, with a *p*-value of < 0.05. The corresponding authors were contacted for clarification in case of any ambiguities.

Due to the number of different factors investigated, and heterogeneity in the measures used, it was not feasible to attempt a meta-analysis of the effect of each factor. Instead, the “best fit” framework synthesis method [43] was used to compare the fit of the data with an existing model of factors affecting health service utilisation. This technique was originally developed for the synthesis of qualitative research, but has since been applied to reviews of quantitative and mixed methods studies [44, 45].

The first author extracted the data from each of the included papers and coded these deductively using the Andersen framework described above [17]. Any data that did not fit any of the headings in the Andersen model headings were to be coded separately under a new theme in a subsequent inductive phase.

To avoid bias in the synthesis and interpretation of results due to pre-conceived ideas about which factors are associated with treatment-seeking, we created a priori definitions with which to categorise the associations found for each factor. These definitions (summarised in Table 3) were created for the purposes of the current review, and are intended to be conservative.

No prior studies were found to guide the operationalisation of these definitions, and therefore the cut-off points chosen are necessarily arbitrary. However, we have tried to be entirely transparent in how these have been applied, and present the full findings and quality ratings in the appendices provided so the reader can examine how the evidence relates to the conclusions drawn.

**Table 3:** Definitions used to grade consistency of evidence when synthesising findings from included studies

Evidence level	Criteria
Good evidence of an association	≥75% of studies that investigated this factor report an association, of which ≥2 (using different datasets) are of good/excellent quality
Good evidence of no association	< 25% of studies that investigated this factor report an association, of which ≥2 (using different datasets) are of good/excellent quality
Inconsistent evidence	25–75% of studies that investigated this factor report an association, of which ≥2 (using different datasets) are of good/excellent quality
Poor quality evidence only	< 2 studies of good/excellent quality (using different datasets) investigated the association between this factor and treatment-seeking for CMD
Not examined	No studies investigated the association between this factor and treatment-seeking for CMD

## Results

### Search results

**Fig. 1:**
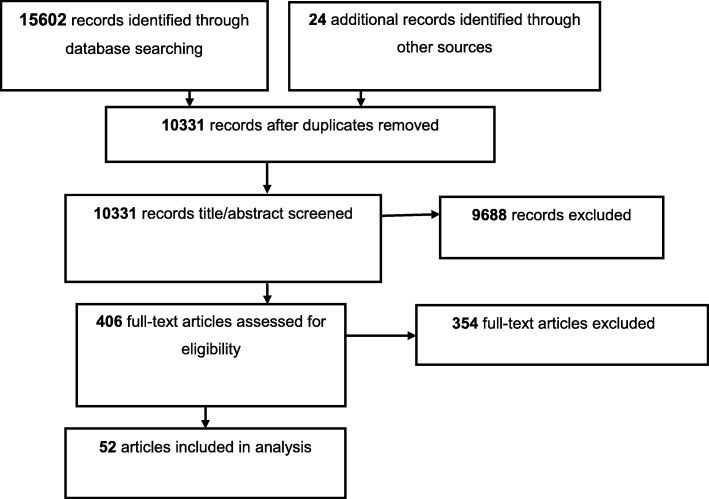
PRISMA flowchart showing selection of studies

Figure 1 summarises the search process. After removing duplicates, 10,331 papers were retrieved. Fifty-two papers were found to meet the criteria at the full text screening stage. Of these, eleven were cohort studies while forty-one were cross-sectional.

Thirty-two (62%) of these studies reported data from North America, nine (16%) from Europe, two (4%) from Australasia, three (6%) from Africa, one (2%) from Asia, two (4%) from Latin America and three (6%) used international data from across world regions.

The study sizes varied considerably, from 56 participants to 18,972 participants with elevated levels of CMD symptoms.

In terms of quality, evidence from one study was rated as poor, evidence from 16 was classified as fair, evidence from 20 was classified as good, and evidence from 15 studies was rated excellent. Additional file 3 presents the characteristics of the included studies.

### Factors associated with health service utilisation for CMD

Table 4 shows the number of studies that investigated each of the factors in the Andersen model.

**Table 4:** Synthesis of associations found between factors in Andersen socio-behavioural model of health service utilisation and treatment-seeking for CMD

Type	Factor	Summary of associations found	Evidence level	No. of studies	Relationship	No. of good quality studies	No. of longitudinal studies	Total sample size	Total number of studies from each region		No. of studies from LMIC
Predisposing factors											
Demographic	Age	Hill-shaped relationship commonly reported, with highest use by middle-aged individuals and lower use by the young and the elderly	Inconsistent	25	Hill-shaped	9	3	33,779	10 1 1	N. America Australasia L. America	1
					Positive/Consistent with hill-shape	2	2	3162	1 1 2	Africa L. America Europe	2
					Mixed	0	1	1926	2	Europe	0
					Null	4	0	6501	2 2 2	Africa N. America Europe	2
	Age of onset	Older age of onset associated with service use in some studies	Inconsistent	3	Positive	2	1	14,640	2	N. America	0
					Other	0	1	1572	1	Europe	0
	Gender	Female gender generally associated with greater service utilisation	Inconsistent	29	Positive (women)	9	5	28,201	10 1 1 1	N. America L. America Africa Europe	1
					Other/mixed	4	2	29,838	3 2 1	N. America Africa Europe	2
					Null	5	2	6380	1 1 2 1 4	Africa Asia N. America Australasia Europe	1
					Negative (women)	1	0	531	1	L. America	1
Social structure	Ethnicity	Caucasian ethnicity commonly associated with greater use than other ethnic groups	Inconsistent	23	Positive (white)	7	2	60,534	11 1	N. America L. America	1
					Positive compared to some ethnicities only	2	0	17,299	3	N. America	0
					Mixed	4	1	36,558	5 1	N. America South Africa	1
					Null	2	1	2658	2 1	N. America Australasia	0
	Immigration status	No clear pattern found	Inconsistent	6	Negative (born abroad)	0	1	2026	1	Europe	0
					Null	2	1	15,056	2 1	N. America Australasia	0
					Mixed	0	0	7687	2	N. America	0
	Marital Status	Being married associated with lower service use in some studies	Inconsistent	18	Negative (married)	5	2	10,367	5 1 1	N. America Europe Australasia	0
					Null	5	1	17,493	1 2 2 2	L. America Africa N. America Europe	3
					Positive (married)	1	1	337	1	N. America	0
					Mixed	2	1	13,590	1 1	N. America Europe	0
	Education	Higher levels of education associated with greater service use in some studies	Inconsistent	20	Positive (higher)	6	4	31,441	6 1 1 1	N. America Europe L. America Africa	2
					Null	7	1	11,377	5 2 1 1	N. America Europe Australasia Africa	1
					Mixed	0	1	2130	1 1	N. America Europe	0
Personality	Conscientiousness	No clear pattern found	Poor	1	Positive	0	1	354	1	Europe	0
	Mastery	No clear pattern found	Poor	1	Mixed	0	1	903	1	Europe	0
	Neuroticism		Inconsistent	2	Positive	1	1	2005	1	Australasia	0
					Null	1	1	102	2	Europe	0
Health beliefs	Prior use of services	Prior use associated with greater use	Good	3	Positive	2	2	13,672	4	N. America	0
					Null	0	0	0	0		0
	Stigma	No clear pattern found	Poor	2	Null	0	0	102	1	Europe	0
					Mixed	1	0	56	1	Asia	1
	Mental Health Literacy	Not investigated		0	N/A	0	0	0			0
Enabling factors											
Assets	Income/ wealth	Most studies did not find any association	Good – no association	11	Null	5	0	7623	3 3 1	N. America Africa Europe	3
					Positive	1	0	7209	1 1	N. America Asia	1
					Mixed	1	0	2510	2	N. America	0
	Employment	Being employed associated with lower use in some studies	Inconsistent	8	Negative (being employed)	2	2	3452	2 2	N. America Europe	0
					Null	3	0	3059	2 1 1	Africa Europe Australasia	2
	Social support	Greater social support linked to use in some studies	Poor	5	Positive	0	1	661	1 1	Europe Africa	1
					Null	1	1	1275	2 1	N. America Europe	0
	Insurance	Having health insurance associated with use in some studies	Inconsistent	7	Positive	2	1	10,393	4	N. America	0
					Null	1	0	956	2	N. America	0
					Mixed	0	0	558	1	N. America	0
Need factors											
Perceived	Self-rated health/perceived need for care	Better self-rated health (/lower perceive need for care) associated with lower service use	Good	8	Negative	2	2	2738	2 1	N. America Europe	0
					Mixed or indirect	2	0	2865	3	N. America	0
					Null	0	1	491	1 1	N. America Asia	1
Evaluated	Symptom severity	Greater severity commonly associated with service use	Inconsistent	16	Positive	5	5	23,165	7 2 1	N. America Europe Australasia	0
					Mixed	1	0	298	1	Europe	0
					Null	3	1	4052	2 2 1	N. America Europe Asia	1
	Chronicity/duration	Longer duration associated with service use	Good	3	Positive	3	0	8603	2 1	N. America Europe	0
	Disability	Greater impairment associated with service use	Good	8	Positive	3	1	4794	1 3 1	N. America Europe Australasia	0
					Mixed/borderline	0	1	2199	2 1	N. America Europe	0
	Comorbid conditions – total	No clear pattern found	Poor	4	Null	1	1	7565	1 1 1	N. America Europe L. America	1
	Non-psychiatric chronic conditions	No clear pattern found	Inconsistent	14	Positive	3	2	17,455	3 1 1	N. America Europe International	1 (combined HIC/LMIC)
					Negative	1	0	220	1	Europe	0
					Mixed	1	0	7460	1 1	N. America Africa	1
					Null	5	1	4567	3 2 1	Europe N. America Australasia	0
	Psychiatric comorbidities (general)	Comorbid mental disorders (in general) associated with service use	Good	6	Positive	5	3	6295	3 2 1	N. America Europe Australasia	0
	Comorbid SUD	No clear pattern found	Inconsistent	8	Positive	3	1	24,189	3	N. America	0
					Negative	1	1	1,1,56	2	N. America	0
					Mixed	0	1	1572	1	Europe	0
					Null	2	1	20,445	2	N. America	0
	Comorbid mood/anxiety disorders	Comorbid mood/anxiety disorders associated with service use	Good	6	Positive	5	4	26,714	3 2	N. America Europe	0
					Null	1	0	102	1	Europe	0
	Other comorbid mental disorders	No clear pattern found	Inconsistent	3	Mixed	3	1	8719	3	N. America	0
	Panic symptoms	Panic symptoms associated with service use	Good	6	Positive	5	2	26,350	4 1	N. America Australasia	0
					Negative	0	0	558	1	N. America	0
	Suicidality	No clear pattern found	Inconsistent	3	Positive	2	0	1646	1 1	N. America Europe	0
					Null	1	1	2864	1	N. America	0
	Somatisation	No evidence of any association	Good – no	2	Null	2	1	1566	2	N. America	0
	Other CMD symptoms	No clear pattern found	Inconsistent	5	Mixed	2	1	15,941	3 2	N. America Europe	0
	Adverse childhood events	No clear pattern found	Poor	4	Positive	0	2	1926	2	Europe	0
					Mixed	1	1	1201	2	Europe	0
Contextual factors											
Place of residence	Urban/rural residence	No evidence of any association	Good – no	7	Null	6	1	16,677	3 2 1 1	N. America Europe Australasia L. America	1
	Country	No clear pattern found	Inconsistent	3	Mixed	2	0	4111	1 1	N. America/Europe N. America/ Australasia	0
					Null	0	0	751	1	N. America	0
	Region (within-country)	No clear pattern found	Inconsistent	3	Positive	1	1	7620	1 1	N. America L. America	1
					Null	1	0	7153	1	N. America	0
Health service factors	Service availability (perceived)	No clear pattern found	Poor	1	Positive	0	1	435	1	N. America	0
	Service accessibility	No clear pattern found	Poor	1	Null	0	0	56	1	Asia	1
	Regular source of care	No clear pattern found	Poor	2	Mixed	1	0	436	2	N. America	0
	Organisation of services (gatekeeper)	No clear pattern found	Poor	1	Negative	1	0	1498	1	N. America	0
	Service capacity/waiting times/opening hours	Not investigated		0	N/A	0	0	0			0
	Resources available	Not investigated		0	N/A	0	0	0			0
	Healthcare policy	Not investigated		0	N/A	0	0	0			0
	Quality of care	Not investigated		0	N/A	0	0	0			0
Social factors	Neighbourhood norms	Not investigated		0	N/A	0	0	0			0

Compared to other factors, we identified the highest number of studies on the association between socio-demographic factors (classified according to the Andersen model as “predisposing” factors) and treatment-seeking for CMD. We also found a large number of studies that investigated symptom severity, symptom profile and comorbidity (termed “need” factors in the Andersen model) as correlates of treatment-seeking. Fewer of the included studies examined enabling factors such as insurance, household wealth and social support. There was a lack of published evidence on some factors implicated by the Andersen model, such as psychological factors (e.g. beliefs and attitudes, classified as “predisposing” factors) and health systems factors (e.g. the availability and accessibility of services).

Almost all of the factors identified were individual rather than contextual level factors. No factors were identified that could not be accommodated by the model.

A summary of findings for each factor group is presented below. For more detailed results see the Additional file 4.

#### Predisposing factors

##### Overall synthesis of findings on predisposing factors

As shown in Table 4, while several trends were identified, no predisposing factors were consistently found to be associated with seeking treatment.

##### Factor-by-factor synthesis of evidence from included studies

*General trends across studies.*

Having sought mental health treatment previously was generally associated with increased likelihood of seeking treatment [46–48]. The relationship between age and health service utilisation for CMD was commonly found to be hill-shaped, with middle-aged respondents most likely to seek treatment [49–68]. Female gender was frequently found to be associated with increased treatment-seeking [46, 47, 49–55, 57–62, 64, 65, 67–77], as was being Caucasian, which represented the majority ethnic group in the context of most of the included studies [46, 47, 49–51, 53, 55, 57–60, 64, 68, 70, 78–87]. Several studies reported that higher education levels were associated with health service utilisation for CMD, although this was not found across all studies [46, 47, 49–51, 53, 55, 57–65, 68, 70, 76, 77]. Being married was negatively associated with treatment-seeking, though it was unclear whether this is due to greater use of services by the never married or by those who are separated or divorced group [46, 47, 49–51, 55, 57–59, 62, 64–68, 70, 73].

*Findings related to other predisposing factors.*

There was mixed evidence with regard to immigration status [46, 48, 68, 73, 82, 88], change in marital status [46, 47, 71], and personality factors [51, 61, 66, 74]. There was limited published evidence available on age of onset, from just three studies, but the findings generally indicated increased likelihood of seeking treatment with later onset [46, 71, 77]. There was also a lack of published evidence on the effect of stigma or other beliefs and attitudes [66, 67].

#### Need factors

##### Overall synthesis of findings on need factors

Need factors were most consistently associated with the use of health services for CMD symptoms across studies, as seen in Table 4.

##### Factor-by-factor synthesis of evidence from included studies

*Consistent findings.*

Five factors were consistently found to be associated with treatment-seeking across studies. These were self-evaluated health status or healthcare needs [50, 53, 67, 68, 70, 74, 83, 86]; duration or chronicity of symptoms [49, 66, 71]; disability or functioning [48, 51, 63, 65, 68, 73, 74, 76]; comorbid mental disorders [46, 49–52, 54, 59, 65, 66, 70, 71, 73, 76, 77, 89–91]; and panic symptoms [46, 51, 52, 71, 91].

*General trends across studies.*

Symptom severity was generally reported to be associated with an increased likelihood of seeking treatment [50, 51, 53, 54, 58, 59, 61, 65, 67, 71, 73, 74, 76, 78, 79, 85].

*Findings related to other need factors.*

There was mixed evidence for an association with suicidality or specific CMD symptoms [46, 47, 51, 52, 59, 63, 66, 71, 76, 77, 85, 91, 92], substance use and non-psychiatric conditions [47, 49–51, 53, 54, 57, 63, 65, 66, 68, 71, 73–75, 93–95] and adverse childhood events [61, 74, 76, 77].

#### Enabling factors

##### Overall synthesis of findings on enabling factors

As indicated in Table 4, there was inconsistent evidence for an association between treatment-seeking for CMD and enabling factors.

##### Factor-by-factor synthesis of evidence from included studies

*Consistent findings.*

The studies included here did not support an association between wealth or income and the use of health services for CMD symptoms [47, 55, 58, 60, 62, 64, 65, 67, 68, 75].

*General trends across studies.*

Some studies indicated a positive association between treatment-seeking and being in employment although this was not found across all studies [50, 51, 58, 62, 65, 73, 75, 76]. Having health insurance was frequently, but not consistently, reported to be correlated with health service utilisation for CMD [47, 49, 50, 53, 60, 63, 68].

*Findings related to other enabling factors.*

There was mixed evidence with regard to social support [50, 61, 66, 68, 75], and limited published evidence available on the effect of having a regular source of care [47, 48].

#### Contextual level factors

##### Overall synthesis of findings on contextual factors

Overall, limited published evidence was found testing the association between contextual level factors and health service utilisation for CMD.

*Consistent findings.*

The studies included here suggest that living in a rural area is not associated with lower rates of treatment-seeking [49, 51, 54, 55, 57, 65, 76].

*Findings related to other contextual factors.*

Few studies compared treatment-seeking between countries or by geographic region within countries, and those that did reported inconsistent findings [49, 57, 59, 78, 96, 97]. There was a dearth of published evidence on the association between the health care environment and utilisation of services for CMD, with just one study on the effect of managed care [53], one on perceived availability of services [50], and one on perceived accessibility of services [67].

### Comparison of evidence from LMIC and HIC

There was a clear discrepancy in the quantity of research identified between high-income and low-and-middle-income countries, with just six of the included studies originating from LMIC and one international study that included data from both HIC and LMIC [93]. Five of the LMIC-only studies were from middle-income countries; two from South Africa [64, 75], and one from Brazil [57], Mexico [69] and China [67]. The only study from a low-income country was from Ethiopia [62].

Evidence from three out of six LMIC studies was rated as good or excellent. On average LMIC studies were smaller than HIC studies, with a mean of 1742 participants with high CMD symptoms, compared to 3374 for HICs.

The LMIC studies identified predominantly reported on the effect of predisposing factors, such as age, gender, and education levels, and on measures of income or wealth.

There was insufficient published evidence from LMIC to compare the factors associated with treatment-seeking for CMD between HIC and LMIC.

### Methodological limitations of included studies

The majority of studies used secondary datasets, which limited the choice of variables to those that are typically collected as part of multi-purpose epidemiological surveys. The frequent use of cross-sectional data also limits our ability to disentangle the direction of causation when associations are found. The majority of studies used multivariate logistic regression models for analysis. The use of hierarchical models, or structural equation modelling that explicitly recognises the potential interactions between some of these factors, may have led to differing conclusions. Although several studies cited the Andersen model to justify their choice of variables, there seems to be little agreement as to how the model should be operationalised and much heterogeneity in the measures used, making it difficult to compare the results across studies. In particular, agreement is needed on how variables indicating level of “need for care” should be measured in the context of CMD, so that is it possible to control for this consistently when investigating whether the use of health services is equitable. Finally, many of the included studies did not correct for multiple testing when investigating multiple associations simultaneously, and as such their findings should be viewed as hypothesis-generating rather than hypothesis-testing.

## Discussion

### Principal findings

This review furthers our understanding of the treatment gap for CMD by summarising patterns of treatment-seeking. Need factors were most consistently found to be associated with treatment-seeking for CMD symptoms. Enabling factors were not found to be consistently associated with treatment seeking for CMD. The evidence on predisposing factors was inconsistent, although there was weak evidence for an association with demographic factors, specifically age, gender, ethnicity, education level and marital status. Finally, the current results suggest that urban or rural residence is not associated with treatment-seeking.

With regard to the second objective, there was insufficient published evidence from LMIC to draw any firm conclusions about whether the factors associated with health service utilisation for CMD differ from high-income countries.

### Strengths and weaknesses

This review has several strengths: It employed a broad search strategy, informed by previous reviews [22–24], since the literature on this topic spans several disciplines with varying terminology. It followed an a priori protocol, had screening verified at multiple stages by a second researcher, and employed a widely recognised theoretical framework to analyse the results. Compared to the most recent review in this area [25], we searched a larger number of databases in order to make the review as comprehensive as possible.

This review adds to previous research by considering the wider category of CMD rather than a single diagnostic category, which several researchers have argued is a more appropriate grouping for community and primary care settings [26, 28–31]. It was also deliberately more liberal in terms of its definition of CMD symptoms, since it is generally accepted that CMD symptoms are better conceptualised as a spectrum rather than a dichotomy between those who meet diagnostic criteria and those who do not [27]. Since conducting full diagnostic interviews in large population studies is often not feasible, it was hoped that this broader definition would lead to the inclusion of studies from a wider range of settings.

Other related reviews have been restricted to young adults [98] or to one country only [99]. While the results reported here are broadly consistent with the findings of these reviews, this study extends previous research by (a) comparing results across settings; (b) including only population-based studies to ensure the generalisability of findings; (c) examining a set of symptoms that typically present together in community settings, making the results a stronger basis for informing interventions at the population level; and (d) separating service utilisation by adults from that of children or adolescents, since in many countries services are delivered separately for these two groups, and decisions regarding treatment-seeking may follow different paths for minors (defined here as those aged under 18).

However, the current review nonetheless has several limitations that must be acknowledged. One is that it was not possible to assess the power of each study to detect an association, meaning that in studies where no association was found with a given factor, this cannot be interpreted with confidence to indicate a lack of association rather than a lack of statistical power. Secondly, it is possible that some studies in which this was not the primary research question may have been erroneously excluded if associations with treatment-seeking were not reported in the title or abstract of the paper. This is more likely to be the case when no associations are found, leading to potential selection bias. For reasons of feasibility, the search was restricted to studies published in English.

We were not able to present data on the amount of variance explained by the factors included in the studies reviewed, since this was not reported in the majority of these studies. Nor was it possible to discuss the confounding factors controlled for in every analysis, due to the large number of studies included. To definitively assess the causal effect of any one factor on treatment-seeking for CMD a meta-analysis of that specific association would be recommended; this was not the purpose of the current review, which set out to summarise associations, not to make causal claims.

The inclusion of multiple measures of CMD symptoms also means that these will not be exactly comparable across studies. Furthermore, when the quality of studies was assessed, we considered the measure of CMD used and the measure of treatment-seeking from the formal health sector; however, due to the number of factors investigated it was not possible to assess the appropriateness of measures used for each of these factors. Finally, as mentioned in the methods section, although the consistency of evidence for each factor was graded according to pre-defined criteria, other ways of operationalising levels of evidence are possible, which could lead to more or less conservative conclusions. Full details of all studies and the criteria applied are presented in the appendices.

### Comparison with previous literature

Our findings are consistent with previous research pointing to need factors as the strongest determinants of health service utilisation for mental disorders [100–103]. This is also consistent with the finding from the World Mental Health Surveys (WMHS) – which included both LMICs and HICs and measured substance use disorders and bipolar disorder as well as CMD – that low perceived need was the most common reason cited for not seeking treatment [104].

The same associations with female gender, middle age, higher levels of education, and being unmarried were found in the WMHS [8].

The fact that the evidence included in the current study did not support an association with economic factors was surprising, given the evidence that socio-economic factors affect the type of provider contacted [63, 68, 77], the quality of care received [63], adherence [105–109] and response to treatment [110, 111]. However, a recent analysis of WMHS data by Evans-Lacko et al. (2017) found that differences in treatment rates in the WMHS by socio-economic status were predominantly accounted for by education rather than income [112].

Thus our findings on treatment-seeking for CMD are largely in keeping with the largest international study of mental disorders and service utilisation to date. The WMHS did not investigate rural/urban residence, or any of the other factors included in the Andersen model besides those listed above.

### Implications

Need factors, reflecting the extent to which CMD symptoms interfere with people’s lives and whether outside help is needed, appear to be central to explaining treatment-seeking behaviour. This suggests that many of those who do not seek care from formal health services for their CMD symptoms fail to do so not because of limited supply, but because of lack of demand for services.

Whether meeting criteria for a disorder is a good indication of a “need for health services” is an ongoing debate in the context of mental health care [113]. The limited demand for interventions for CMD, compared to the number of people who meet criteria for CMD, can be conceptualised as a lack of education or awareness about mental health issues, indicating a need for information, education and communication campaigns. On the other hand, it may be an indication that current diagnostic categories are overly broad, and include a large number of people who do not require formal medical care. Patel (2014) has argued that current prevalence estimates should not be regarded as the number of individuals in need of care, since a large proportion of these individuals do not require formal interventions through the health system [31]. Measures of functioning or quality of life may represent better indicators of “need for care” than meeting diagnostic criteria (it is notable that the latter concept was not investigated by any of the studies included here).

Patel’s argument that increasing the supply of mental health services will not alone make a substantial impact on the treatment gap for mental disorders is supported by the current findings that; (a) lack of perceived need is a major determinant of failure to seek help from health services, and (b) that enabling factors do not appear to be a major determinant of treatment-seeking (discussed below). Many individuals with less disabling symptoms are likely to view informal support – such as social interventions in the community, or advice on self-care, listed at the bottom of the World Health Organization (WHO) Service Organization Pyramid [114] – as more appropriate for their needs. As such, encouraging these individuals to seek care through the health system may not be the best use of resources.

The lack of evidence for an association between enabling factors and health service utilisation, even in settings with weak public health systems, such as South Africa, and without universal health coverage, like the USA, was surprising. Of course, absence of evidence is not proof of a lack of association, especially given that the studies included here were not explicitly powered to detect this relationship. There is also the potential for information bias, given the sensitivity of financial topics, since most studies used self-reported data.

However, the hypothesis that economic factors do not play a major role in determining whether people with CMD initially seek care from health services is backed up by findings from Evans-Lacko et al. (2017) [112], as well as Andrews et al. (2001), who found no association at the ecological level with health spending or out-of-pocket costs [112, 115]. Furthermore, Andrade et al. (2014) found that attitudinal barriers (most commonly, wanting to handle the problem alone) were reported much more often than structural barriers (which are linked to enabling factors), with the exception of severe cases. It is possible that the inclusion in this review of individuals with milder conditions, for whom low perceived need primarily inhibits treatment-seeking, might be obscuring the real impact of enabling factors such as cost and travel distance on the sub-group with severe CMD, who are most in need of care. Future research could usefully examine the extent to which supply side factors such as the availability, affordability and accessibility of care affect service utilisation by those with the most severe needs.

If equitable access to health care is defined as equal utilisation by those with equal need for care [116], then there is some evidence pointing to the need to target underserved groups such as men, ethnic minority groups, the elderly and young adults, at least in HIC. However, the extent to which need factors such as symptom severity and disability were controlled in these analyses varied between studies, so we cannot definitively rule out the possibility that these differences can be explained by variability in need for treatment.

Attempts to address these inequities have been made in HIC through strategies such as enhancing cultural competence in mental health services [117] and targeting underserved groups through social marketing [118], with some success [119, 120]. Evaluations of these interventions typically measure adherence/attrition, patient satisfaction or attitudes towards seeking care rather than treatment-seeking behaviour, so their effectiveness in reducing mental health care inequities is still to be determined.

Regarding geographic location, some studies indicate that this may affect the type of provider chosen and the quality of care received [54, 121]. It is possible that the initial decision of whether or not to seek treatment is made independently of location of residence, but the subsequent decision of where to seek treatment, and the health system’s response, is influenced by geography. This wants further investigation (see “Unanswered questions and future research”, below).

Finally, although it was not the topic of this review, there was some evidence to suggest that the factors associated with health service utilisation for CMD may vary between the specialist and generalist sectors [64, 77, 87], which has been highlighted in other studies [100, 112]. This warrants further investigation as it has important implications for service planning. Thornicroft and Tansella (2013) advocate a stepped care model of mental health services, with the majority of services delivered through primary care in low-resource settings [122]. However, it remains to be investigated which balance leads to the most equitable use of services for CMD, and whether some groups are more likely to seek treatment through primary care in LMIC.

### Unanswered questions and future research

This review identified three major gaps in our knowledge: Firstly, a lack of research from LMIC; secondly, a dearth of research on contextual factors, particularly health systems factors; and thirdly, an absence of studies that are explicitly powered to test associations between the factor of interest and treatment-seeking for CMD.

The first of these gaps directly relates to the second objective of this review. Although we have drawn some tentative conclusions above, the generalisability of these findings to LMIC is questionable at best, since nearly 90% of the studies identified were from high-income countries. In contrast, 85% of the world’s population is expected to live in LMIC by 2030 [123], making this is an extremely important omission.

Not only was there a noticeable lack of population-based studies from LMIC, but those studies that were identified were less consistent in their findings than those from HICs. This may be in part due to the reduced statistical power of studies from areas where treatment rates are low, meaning that larger sample sizes are needed to detect an association. More research is urgently needed in LMIC – especially in those countries for which no population-based studies were identified – to determine whether the same factors are associated with treatment-seeking for CMD in non-Western settings, using large enough samples to detect an association.

Secondly, there was also a notable lack of published evidence on several contextual factors, in particular health systems factors that are likely to affect treatment-seeking. This includes the availability of services, the geographical accessibility of those services, and characteristics of services such as opening times, which are central to several models of access to health care [15, 124, 125]. This is a crucial gap, as such evidence could usefully inform service planning to expand access to care.

The extent to which distance affects treatment-seeking has particular relevance to debates around decentralisation and integration of mental health care [126]. Facility-based studies have pointed to distance and travel time as a potentially important determinant of health service utilisation [127–131], which contrasts with the lack of evidence supporting an association with urban/rural residence found in this review. However, these studies cannot disentangle geographic differences in prevalence from differences in treatment seeking behaviour. Furthermore, unless they assess the use of *all* health facilities in a given area – both public and private – it is not clear if distance affects whether affected individuals seek any care, or if it merely influences the choice of provider among those who do decide to seek treatment. This review showed that there is a lack of population-based data on the influence of geographic accessibility on the uptake of health services for CMD, with the exception of crude comparisons of rural and urban areas, for which no association was found with treatment-seeking.

Finally, none of the studies included here justified their sample size with regard to the relationship between treatment-seeking and the factors investigated. It is therefore possible that the lack of associations identified in some of the studies included here are the result of under-powered studies, rather than a genuine lack of association. To build the evidence base in this area and confirm the hypotheses generated by the current review, future studies should ensure that they have sufficient statistical power to detect an association with the factors investigated.

## Conclusions

This review found that the set of factors most consistently associated with formal health service utilisation for CMD among the adult population were need factors, with inconsistent evidence of an association with predisposing factors – specifically demographic factors – and little evidence to support an association with enabling factors. Health system factors, such as the availability and accessibility of services, are under-researched in population-based studies. Research in low and middle-income countries is urgently needed to enhance our understanding of treatment-seeking for CMD in order to inform efforts to expand access to effective interventions and increase health service utilisation for CMD by those with greatest need for care.

## Additional files

Additional file 1: PRISMA 2009 Checklist. (DOC 63 kb)

Additional file 2: Search strategy (Medline). (DOCX 14 kb)

Additional file 3: Characteristics of included studies on factors associated with health service utilisation for CMD. (DOCX 38 kb)

Additional file 4: Detailed results by factor. (DOCX 141 kb)

## Abbreviations

CIS-R: Clinical Interview Schedule - Revised
CMD: Common mental disorder
HIC: High-income countries
ICD-10: International Statistical Classification of Diseases and Related Health Problems - 10th Revision
LMIC: Low- and middle-income countries
MMAT: Mixed-Method Appraisal Tool
PHQ-9: Patient Health Questionnaire-9
PRISMA: Preferred Reporting Items for Systematic Reviews and Meta-Analyses
WHO: World Health Organization
WMHS: World Mental Health Surveys
