# Supplementary material for: Factors associated with health service utilisation for common mental disorders: a systematic review
Source: BMC Psychiatry. 2018 Aug 22;18:262. doi: 10.1186/s12888-018-1837-1 (PMC6104009; doi:10.1186/s12888-018-1837-1)
Supplement: Supplementary file 2 — Search strategy (Medline). (DOCX 14 kb) [file 12888_2018_1837_MOESM2_ESM.docx]

**Additional file 2** Search strategy (Medline)

1. (depression or (depressive adj2 disorder$)).ti,ab.

2. depressive disorder/

3. CMD.ti,ab.

4. somatoform.ti,ab.

5. (common adj2 (mental adj2 disorder$)).ti,ab.

6. MUS.ti,ab.

7. (medically adj2 unexplained).ti,ab.

8. (pathway* adj2 care).ti,ab.

9. barrier$.ti,ab.

10. access$.ti,ab.

11. ((health adj2 service$) or healthcare or (health adj2 care)).ti,ab.

12. (access$ adj4 ((health adj2 service$) or healthcare or (health adj2 care))).ti,ab.

13. (barrier$ adj4 ((health adj2 service$) or healthcare or (health adj2 care))).ti,ab.

14. "Delivery of Health Care"/ut [Utilization]

15. Health Services/ut [Utilization]

16. coverage.ti,ab.

17. utili#ation.ti,ab.

18. (utili#ation adj4 ((health adj2 service$) or healthcare or (health adj2 care))).ti,ab.

19. (coverage adj4 ((health adj2 service$) or healthcare or (health adj2 care))).ti,ab.

20. "use".ti,ab.

21. ("use" adj4 ((health adj2 service$) or healthcare or (health adj2 care))).ti,ab.

22. "Patient Acceptance of Health Care"/

23. Health Services Accessibility/

24. Help-Seeking Behavior/

25. Healthcare Disparities/

26. help?seek$.ti,ab.

27. (service$ adj2 contact).ti,ab.

28. 8 or 12 or 13 or 14 or 15 or 18 or 19 or 21 or 22 or 23 or 24 or 25 or 26 or 27

29. Anxiety Disorders/

30. (anxiety adj2 disorder$).ti,ab.

31. 1 or 2 or 3 or 4 or 5 or 6 or 7 or 29 or 30

32. 28 and 31

33. limit 32 to humans

34. limit 33 to "all adult (19 plus years)"

35. limit 34 to english language
