# Supplementary material for: Factors associated with health service utilisation for common mental disorders: a systematic review
Source: BMC Psychiatry. 2018 Aug 22;18:262. doi: 10.1186/s12888-018-1837-1 (PMC6104009; doi:10.1186/s12888-018-1837-1)
Supplement: Supplementary file 3 — Characteristics of included studies on factors associated with health service utilisation for CMD. (DOCX 38 kb) [file 12888_2018_1837_MOESM3_ESM.docx]

| **Study** | **Country** | | **Study design** | | | **Population** | **CMD measure** | **Outcome** | | **Factors associated** | **Andersen coding** | **Sample size** | **Response rate (overall)** | | | **Sampling strategy appropriate** | | **Sample representative of population** | | **Measures appropriate** | | **Accept-able response rate** | | **Over-all score** | |  |
| --- | --- | --- | --- | --- | --- | --- | --- | --- | --- | --- | --- | --- | --- | --- | --- | --- | --- | --- | --- | --- | --- | --- | --- | --- | --- | --- |
| Alegría et al. (2008) | USA | | Cross-sectional | | | Combined 3 nationally representative samples; one of the general adult population, 1 of the adult black population and 1 of the adult Latino and Asian population | 12 month major depression or dysthymia (CIDI) | 12 month visit to a specialist or general medical provider for mental health reasons | | Ethnicity | Predisposing (ethnicity) | 1,082 with depression/ dysthymia | 70.9%-77.7% | | | Strategy appropriate but no justification of sample size | | Yes (when weighted) | | Yes | | Yes | | **** | |  |
| Andersson et al. (2013) | South Africa | | Cross-sectional | | | 18-40 year olds (random population-based sample) | Lifetime depression (DSM-IV criteria) | Lifetime help-seeking from healthcare staff for emotional reasons | | Age (18-29 less likely), comorbid TB, social support. null: sex, employment, income, comorbid HIV (p=0.07). Sex interacted with age; sex differences in older but not younger groups. | Predisposing (age, null: sex), enabling (social support, null: employment, income), need (comorbid TB, null: HIV) | 307 with lifetime depression | Not reported | | | Strategy appropriate. Sample size justified but not clear what question they're addressing with this | | Yes (representative of 18-40 year olds) | | Yes (though lifetime recall questionable) | | Unknown | | * | |  |
| Angst et al. (2010) | Switzerland | | Prospective cohort | | | Adults with depression, bipolar, anxiety, panic, neurasthenia and insomnia (SCL-90-R), stratified subsample by severity score | Depression/anxiety (SPIKE - DSM-III or DSM-IV criteria, but including subthreshold cases) | 12 month use of health services (generalist and specialist) for MH symptoms | | For depression: sex, subjective distress, childhood family problems (null: work impairment, social impairment, mastery, comorbidities). For GAD/panic attacks: subjective distress, work impairment (null: sex, social impairment, mastery, childhood family problems, comorbidities). Being above threshold for diagnostic criteria not associated | For depression: predisposing (sex), need (subjective distress, childhood family problems, null: work impairment, social impairment, "mastery", comorbidities, meeting full diagnostic criteria). For GAD/panic attacks: predisposing (null: sex), need (subjective distress, work impairment, null: social impairment, mastery, childhood family problems, comorbidities, meeting full diagnostic criteria) | 323 with depression, 192 with panic disorder and 388 with GAD (not necessarily meeting diagnostic criteria) | Initial response rate 62% (men; 66%, women; 58%). 62.1% retained in the study for the full 20 years (dropouts did not differ significantly on most characteristics). Refusers had lower educational levels but otherwise no socio-demographic differences. | | | Males and females sampled through separate means, sampling frame somewhat unclear for females (addresses provided "by the communities"). Total sample size not justified but there is reference to stratification and over-sampling of those at risk to give "a sufficient number for further analyses | | Sample representative of canton of Zurich | | Yes | | Borderline - women only 58% | | ** | |  |
| Ault-Brutus (2012) | USA | | Cross-sectional | | | Adults aged 18-54 (from nationally representative sample) | 12-month mood and/or anxiety disorder (CIDI - DSM-III-R or DSM-IV criteria, depending on wave) | 12 month visit to a health professional for MH reasons | | Race/ethnicity - black people less like to use services than white/Latino people (not mediated by SES, partially mediated by perceived need) | Predisposing (ethnicity), mediated by need (perceived) | 2127 | 82.4% (wave 1), 70.9% (wave 2) | | | Strategy appropriate but no justification of sample size | | Yes (representative of 18-54 yr-olds) | | Yes | | Yes | | *** | |  |
| Ault-Brutus & Alegria (2016) | USA | | Cross-sectional | | | White, black or Latino adults aged 18–54 (from nationally representative sample) | 12 month mood/anxiety disorder (CIDI - bipolar excluded) | 12 month visit to a specialist or generalist medic for MH reasons | | Ethnicity not associated with perceived need, but among those with perceived need for care there were ethnic disparities - blacks and Latinos less likely to receive treatment than whites - in 2001/2003 but not 1990/1992 | Predisposing (ethnicity) interacts with need (perceived) | 2127 with mood/anxiety disorders | 82.4% and 70.9% | | | Strategy appropriate but no justification of sample size | | Yes (representative of 18-54 year olds who are white/black/Latino) | | Yes | | Yes | | *** | |  |
| Bauldry & Szaflarski (2017) | USA | | Cross-sectional (part of a cohort study but current analyses used cross-sectional data) | | | Adult civilian, non-institutionalized population (from nationally representative sample) of European, African/Afro-Caribbean, Asian/Pacific Islander, Hispanic, or Puerto Rican background (excluded those of Canadian, Australian, Native American, or Middle Eastern origins) | 12 month mood/anxiety disorder (AUDADIS-IV - DSM-IV criteria - bipolar excluded) | 12 month disorder-specific health service use (not 100% clear that this excluded non-health service providers though - separate question added for cross-sectional analysis that's less specific that longitudinal questions) | | In adjusted model, first generation immigrants less likely to use services for mood disorders but doesn't reach significance for anxiety disorders. Second generation immigrants no different from non-immigrants. People of African and Hispanic origin have lower odds of utilizing mental health care for either mood or anxiety disorders than people of European origin. Acculturation (identify dimension) increases treatment-seeking - still significant in adjusted model for mood but not anxiety disorders or for any disorders among people of European origin | Predisposing (immigration status, ethnicity, acculturation) interacts with need (disorder type) | 3,230 (mood), 4,239 (anxiety) | 89% (wave 1), 86.7% (wave 2) | | | Strategy appropriate but no justification of sample size | | Yes (representative of main ethnic groups) | | CMD measure appropriate but outcome not clearly defined | | Yes | | ** | |  |
| Boerema et al. (2016) | Netherlands | | Cross-sectional | | | General adult population (excluding those with low level of Dutch language) | MDD (CIDI 2.1) | 6 month contact with a health care professional for MH reasons | | Duration of symptoms (longer - more treatment-seeking), personal stigma (less - more treatment-seeking). Null: age, partner status, severity (X2?), comorbid anxiety, comorbid physical complaints, neuroticism, loneliness, perceived stigma | Predisposing (personal stigma, null: age, marital status, neuroticism, perceived stigma), enabling (null: loneliness*), need (duration, null: comorbid anxiety, comorbid physical complaints) (*could be seen as enabling/need/predisposing) | 102 with MDD | 28% (of those who scored positive on K-10, response rate to K-10 not reported) | | | Strategy appropriate but no justification of sample size | | Yes (representative of those who speak Dutch) | | Yes | | No | | *** | |  |
| Bucholz & Dinwiddie (1989) | USA | | Prospective cohort | | | Adults from community survey | Elevated symptoms of depression/dysthymia (DIS - not necessarily meeting full DSM-III criteria) | 12 month discussion of depressive episode with a doctor | | Comorbid psychiatric conditions (OCD/panic disorder more likely, substance abuse less likely). Mania, schizophrenia, phobia and somatisation not associated (antisocial personality disorder approached significance p=0.09) | Need (some comorbid psychiatric conditions, null: other comorbid psychiatric conditions) | 218 | 80% at baseline, 85% follow-up | | | Strategy appropriate but no justification of sample size | | Sample representative of St. Louis | | Yes | | Yes | | **** | |  |
| Bucholz & Robins (1987) | USA | | Cross-sectional (part of a cohort study but current analyses used cross-sectional data) | | | General adult population (includes those in institutions) | 18m depression/dysthymia (DIS - DSM-III criteria) | 12m discussion with a doctor about the depressive symptoms | | In fully adjusted model, appetite symptoms, worsening of physical health, being female, being separated/widowed, previous use of specialty mental health services and not using ER as usual source of care were associated with treatment-seeking. Null: prior consultation with a doctor about somatic symptoms, race, education, recent change in marital status, household income, health insurance, having a usual source of care | Predisposing (gender, marital status, null: ethnicity, change in marital status), enabling (null: education, household income, health insurance, usual source of care), need (specific symptoms: appetite, worsening physical health, previous speciality service use, not using ER as usual care, null: prior contact with doc for somatic symptoms) | 218 with depression/dysthymia | 75% to 80% (varied by site) | | | Strategy appropriate but no justification of sample size | | Yes | | Yes | | Yes | | **** | |  |
| Burnett-Zeigler et al. (2012) | USA | | Prospective cohort | | | Adults with 12-month major depression or dysthymia, persistent symptoms and no prior depression treatment (from nationally representative sample) | 12 month depression /dysthymia (AUDADIS-IV - DSM-IV criteria) | Use of health services for MH reasons (including inpatient and emergency care) between baseline and follow-up (approx. 3 years) | | Gender, substance use, race/ethnicity, marriage status, education, self-rated health, anxiety disorders | Predisposing (sex, ethnicity, marital status), enabling (education), need (substance use, self-rated health, anxiety disorders) | 337 | 81.0% at baseline, 86.7% at follow-up | | | Strategy appropriate but no justification of sample size | | Yes (representative of those with persistent symptoms and no prior treatment) | | Yes | | Yes | | *** | |  |
| Carragher et al. (2010) | | USA | | Cross-sectional | Non-institutionalised adults with a lifetime diagnosis of MDD (from nationally representative sample) | | Lifetime MDD (AUDADIS-IV - DSM-IV criteria) | | Lifetime use of health services for depressive symptoms (includes inpatient and emergency care) | Sex, ethnicity, education, age, income, insurance, number of depressive episodes, some comorbid medical conditions (high blood pressure, arthritis), comorbid mood/anxiety disorder. Not associated: marital status, region, urbanicity, comorbid alcohol problems or personality disorder, other medical conditions (liver problems, heart/artery problems, stomach problems) | Predisposing (sex, ethnicity, age, null: marital status), enabling (education, income, insurance, null: region, urbanicity), need (number of depressive episodes, some comorbid medical conditions, comorbid mood/anxiety disorder, null: comorbid alcohol problems, personality disorder, other medical conditions) | | | 7153 | 81% | | Strategy appropriate but no justification of sample size | | Yes | | Yes (though lifetime recall questionable) | | Yes | | *** | |
| Chartrand et al. (2012) | | USA | | Prospective cohort | Adult non-institutionalized population | | MDD (AUDADIS-IV - DSM-IV criteria) | | Use of any health services for MH reasons since start of study (approx. 3 years) | Suicidality (behaviour and ideation) associated in univariate analyses but not adjusted model (except hospitalisation). In unadjusted analyses, gender, age, ethnicity, marital status, region, education, comorbid mental disorder and depression severity were all associated | Predisposing (unadjusted: gender, age, ethnicity, marital status), enabling (education), need (null: suicidality, except being hospitalised, unadjusted: comorbid mental disorder, severity), contextual (region) | | | 2864 with MDD | 70.20% | | Strategy appropriate but no justification of sample size | | Yes | | Yes | | Yes | | **** | |
| Chen (2012) | | China | | Cross-sectional | Urban residents aged 18-70 from household survey | | Psychological distress (K10>=20) | | Help seeking for emotional reasons in the previous 12 months (separated by health professionals, informal support and alternative services) | Concerns about affordability negatively associated, no association for concerns about accessibility, refusal to recognise need, lack of trust, embarrassment or stigma, symptom severity, self-rated physical health, age, gender or marital status | Predisposing (null: age, gender, marital status, stigma/embarrassment, lack of trust in professional services), enabling (affordability concerns, null: accessibility concerns), need (null: severity, recognition of need, self-rated physical health) | | | 56 with high distress scores | 51% | | Strategy appropriate but no justification of sample size | | Yes (representative of urban population aged 18-70 in Beijing) | | Double-check K-10 properties. Screener not diagnostic tool | | No | | ** | |
| Chen (2013) | | USA | | Cross-sectional (part of a cohort study but current analyses used cross-sectional data) | Adult non-institutionalized population (original sample included under-18s but excluded in current study) | | MDE (CIDI - DSM-IV criteria) | | 12 month use of health services for MH reasons | Comorbid substance dependence (after adjusting for sociodemographic characteristics). Multiple comorbid SUDs associated with greater treatment-seeking | Need (comorbid SUD) | | | 18,972 with MDE | Weighted response rates for household screening and for interviewing: 91.3%, 76.2%, respectively (2005)90.6%, 74.2% (2006)89.5%, 73.9% (2007)89.0%, 74.4% (2008)88.8%, 75.7% (2009)88.8%, 74.7% (2010) | | Strategy appropriate but no justification of sample size | | Yes | | Yes | | Yes | | **** | |
| Demyttenaere et al. (2006) | | Belgium, France, Germany, Italy, the Netherlands and Spain | | Cross-sectional | Community-dwelling adults (nationally representative sample) | | Depression (CIDI 3.0) | | 12 month help-seeking from health services for MH reasons | Comorbid painful physical symptoms (reduces treatment-seeking) | Need (comorbid pain) | | | Weighted sample of 5489 (of whom 220 had 12 month MDE) | 61.2% (but ranges from 45.9% in France to 78.6% in Spain) | | Strategy appropriate, sample size justified for cross-country comparisons of treatment gap, not effect of pain | | Yes | | Yes | | Borderline (acceptable in some countries but not others) | | *** | |
| Demyttenaere et al. (2008) | | Belgium, France, Germany, Italy, the Netherlands and Spain | | Cross-sectional | Community-dwelling adults (nationally representative sample) | | Anxiety (CIDI 3.0) | | 12 month help-seeking from health services for MH reasons | Comorbid painful physical symptoms (increases treatment-seeking but doesn't reach stat. sig.) | Need (comorbid pain) | | | Weighted sample of 5489 (of whom 280 had 12 month anxiety disorder w/o comorbid mood disorder) | 61.2% (but ranges from 45.9% in France to 78.6% in Spain) | | Strategy appropriate, sample size justified for cross-country comparisons of treatment gap, not effect of pain | | Yes | | Yes | | Borderline (acceptable in some countries but not others) | | *** | |
| Fortney et al. (1998) | | USA | | Prospective cohort | Adults with current depression symptoms | | Depressive disorder - major depression/dysthymia/subthreshold depression (Burnam depression screener) | | 6 month use of health services for depression, or in which depression was diagnosed/mentioned in notes/antidepressant prescribed (not clear whether visits for other MH reasons included or not) | Age, gender, employment status, depression severity, and psychiatric comorbidity, "Expected maximum utility of sector choice", insurance coverage and availability. Null: ethnicity, living alone, education, social support, perceived need for depression treatment, medical comorbidities | Predisposing (age, sex, null: ethnicity), enabling (employment, insurance, availability?, "expected utility of sector" Null: education, social support, living alone), need (severity, psychiatric comorbidity, null: perceived need, medical comorbidity) | | | 435 | 70.5% (then 73.9% of depressed sub-sample) | | Strategy appropriate but no justification of sample size | | Representative of those with telephones and no comorbidities | | Screener only | | Yes | | ** | |
| Gabilondo et al. (2011) | | Spain | | Cross-sectional | Community-dwelling adults (from nationally representative survey) | | 12-month major depressive episode (CIDI) | | 12-month use of services for MH reasons - includes non-health sector providers but reported separately | Unemployment/not working due to disability, comorbid mental disorders. Null: age (doesn't reach significance), sex, education, marital status, urbanicity, income, severity, chronic general medical conditions | Predisposing (null: age, sex, marital status), enabling (employment*, null: education, urbanicity, income), need (comorbid mental disorders, disability*, null: severity, chronic physical conditions), *same measure used | | | 247 | 78.60% | | Strategy appropriate, sample size justified for cross-country comparisons of treatment gap, not sub-group analyses | | Yes | | Yes | | Yes | | **** | |
| Galbaud du Fort et al. (1999) | | Canada | | Cross-sectional | Adults (aged 18+) from household survey | | Lifetime depressive illness (DIS) | | Lifetime discussion of symptoms with a doctor or "other professional" (not defined) | Sex, age at first onset, duration of illness, bereavement, specific psychiatric symptoms and comorbid psychiatric disorders; psychomotor retardation, suicidal ideation, mania, panic disorder, drug abuse/dependence (latter reduced chances whereas others increased chances of seeking treatment). Some interactions (suicidal ideation with age at first onset, comorbid OCD and age at first onset, duration with weight loss and alcohol abuse). Null: number of depressive symptoms, number of comorbid diagnoses, specific symptoms (hypersomnia, weight gain, loss of appetite, trouble concentrating, guilt, disinterest in sex, thoughts of death*), somatisation, comorbid schizophrenic disorders or eating disorders | Predisposing (sex, age of onset), need (duration, comorbid disorders, suicidal ideation, psychomotor retardation, drug abuse). Not clear how to classify bereavement - could be seen as predisposing, enabling or need | | | 1348 | 71.60% | | Strategy appropriate but sample size not justified | | Yes | | Outcome measure ambiguous (plus lifetime recall questionable) | | Yes | | *** | |
| González et al. (2010) | | USA | | Cross-sectional | Combined 3 nationally representative samples; one of the general adult population, 1 of the adult black population and 1 of the adult Latino and Asian population. Current study included only Mexican, Puerto Rican, Caribbean black, African American, and non-Latino white respondents | | 12 month MDD (CIDI - DSM-IV criteria) | | 12 month use of pharmacotherapy or psychotherapy | Mexican American/African Americans less likely to receive treatment than whites (but difference not stat. sig. for Puerto Ricans or Caribbean blacks in adjusted analyses). Age also associated (35-64 yr-olds most likely to use care), and health insurance. Null: education, household income, gender (approached significance, 0.05>p<0.01) | Predisposing (ethnicity, age, null: gender), enabling (insurance, null: education, income) | | | 1307 with MDD | 72.30% | | Strategy appropriate but no justification of sample size | | Yes (representative of main ethnic groups) | | CMD measure appropriate but outcome excludes consultations that didn't result in treatment | | Yes | | ** | |
| Gwynn et al. (2008) | | USA | | Cross-sectional | City-dwelling non-institutionalized adults (aged 20 years or older) | | 12 month MDD or anxiety (CIDI - DSM-IV criteria) | | 12 month consultation with a MH specialist or use of pharmacotherapy | For depression: previous diagnosis, symptoms limiting ability to work, being born in the USA. For anxiety: having a regular source of medical care, symptoms limiting ability to work. Null associations not reported. Education and nativity associated with lack of diagnosis (more educated - less likely to be diagnosed, foreign-born - less likely to be diagnosed) | DEPRESSION: Predisposing (nativity, previous diagnosis*), need (work impairment) *Could be categorised as need variable. ANXIETY: enabling (regular source), need (work impairment) | | | 145 with MDD, 73 with anxiety | 55% | | Strategy appropriate, sample size powered for accuracy of prevalence measure | | Yes (representative of urban residents aged 20+) | | CMD measure appropriate but outcome excludes consultations in generalist settings where pharmacological treatment not prescribed) | | No | | ** | |
| Hailemariam et al. (2012) | | Ethiopia | | Cross-sectional | Adults aged 18+ (from nationally representative survey) | | 12 month depression (WHO-CIDI - ICD-10 criteria) | | 12 month use of health services for depressive symptoms | Educational status. Urban-rural, age, marital status, employment status, income and gender differences didn't reach statistical significance in multivariate analyses | Predisposing (null: gender, age, marital status), enabling (education, null: urbanicity, employment, income) | | | 4925 (449 with depression) | 99% | | Strategy appropriate but no justification of sample size | | Yes | | Yes | | Yes (suspiciously high…) | | **** | |
| Hamalainen et al. (2008) | | Finland | | Cross-sectional | Adults aged 30+ (from nationally representative sample) | | Major depressive disorder or anxiety disorders (CIDI) | | 12 month health service use for mental health reasons | For MDD: severity, perceived disability, psychiatric comorbidity, specific symptoms (feelings of inferiority, suicide plans) and living alone. Null: sex, age, employment, education, rurality, somatic disorders, parents' psychiatric problems. For anxiety: perceived disability, psychiatric comorbidity, younger age, and parent's psychiatric problems. Null: sex, marital status, employment status, education, rural/urban residence, somatic disorders | Differences by disorder (anxiety vs. depression). For depression, need only (severity, perceived disability, psychiatric comorbidity, feelings of inferiority, suicidal plans). For anxiety, predisposing (age, parent's psychiatric problems) and need (perceived disability, psychiatric comorbidity). Null associations not clear - only amalgamated results reported and statistical tests refer to differences in service type | | | 298 | 75% | | Strategy appropriate but no justification of sample size | | Yes (representative of adults aged 30+) | | Yes | | Yes | | *** | |
| Hankerson et al. (2011) | | USA | | Cross-sectional | Non-Hispanic black and white adults (from nationally representative sample) | | 12 month MDD (DSM-IV criteria) | | 12 month receipt of any mental health treatment (includes inpatient and emergency services) | Race/ethnicity | Predisposing (race/ethnicity) | | | 1866 | 81% | | Strategy appropriate but no justification of sample size (though check Grant et al., 2004) | | Representative of 2 main ethnic groups, excludes others | | Yes | | Yes | | *** | |
| Issakidis & Andrews (2002) | | Australia | | Cross-sectional | People who reported anxiety as their principal complaint (from nationally representative sample) | | Anxiety (CIDI) (restricted to those for whom anxiety was principal complaint) | | 12 month consultation with a health professional for MH reasons | Age, marital status, disability, neuroticism, disorder type, severity, number of comorbid mental disorders. Sex, education, employment status, ethnicity, urbanicity, and physical disorder not associated | Predisposing (age, neuroticism, marital status, null: sex, ethnicity), enabling (null: urbanicity, education, employment), need (disability, disorder type, severity, comorbid mental disorders, null: physical disorders) | | | 2005 | 78.10% | | Strategy appropriate but no justification of sample size | | Excludes those for whom anxiety symptoms were not principal complaint | | Yes | | Yes | | *** | |
| Iza et al. (2013) | | USA | | Prospective cohort | Adults aged 18+ (from nationally representative sample) | | Anxiety disorders (DSM-IV criteria) | | Lifetime treatment-seeking for anxiety symptoms from doctor, psychologist, therapist, counselor "or any other service provider" | Disorder type (panic highest, phobia lowest). Associated for all disorders: age at onset, change in marital status, prior MH treatment, comorbid mood disorders. Associated for some disorders: sex (GAD only), ethnicity (black - reduced HSE for phobia), education (GAD and phobia only), marital status (never married - increased HSU for social anxiety). Null for all: nativity, SUD/SUD treatment, (all results are after adjusting for sex, race, nativity and age at onset) | Predisposing (sex, ethnicity, prior MH treatment*, age at onset, null: nativity), enabling (education, marital status), need (disorder type, comorbid mood disorders, null: SUD). *Could be classified as need factor. Note differences by disorder type | | | 13292 | 86.7% (for wave 2), 70.2% (from original sample - reported elsewhere) | | Strategy appropriate but no justification of sample size (though check Grant et al., 2003) | | Yes | | Outcome measure ambiguous (plus lifetime recall questionable) | | Yes | | *** | |
| Keyes et al. (2008) | | USA | | Cross-sectional | Non-Hispanic white or black adults aged 18+ residing in households and group quarters | | Lifetime anxiety disorders (AUDADIS-IV - DSM-IV criteria) (mood and substance use disorders also measured but presented separately) | | Lifetime use of health services for MH reasons | White respondents were significantly more likely than black respondents to use services in all models | Predisposing (ethnicity) | | | 32752 | 81% | | Strategy appropriate but no justification of sample size | | Yes (representative of white and black populations) | | Yes (though lifetime recall questionable) | | Yes | | ** | |
| Lee et al. (2011) | | USA | | Cross-sectional | Community-dwelling adults with lifetime major depression or anxiety disorders (from nationally representative survey) | | Lifetime major depression and anxiety disorders (AUDADIS-IV - DSM-IV criteria) (other disorders measured but reported separately) | | Lifetime use of services for specific disorders (12m also measured) - includes inpatient and emergency services | Race/ethnicity (after adjusting for socioeconomics and years of residency in the US) - for depression and anxiety, but different patterns for each | Predisposing (ethnicity) | | | 6624 (depression), 7241 (anxiety disorders) | 81% | | Strategy appropriate but no justification of sample size | | Yes | | Yes (though lifetime recall questionable) | | Yes | | *** | |
| Lee et al. (2014) | | USA | | Cross-sectional | Community-based sample of adults aged 18+ (excluding Native Americans) | | Lifetime depressed mood or anhedonia (AUDADIS-IV - DSM-IV criteria A) | | Lifetime use of health services for mood problems | Sub-type of depression (severity but also differences between "cognitive" and "psychosomatic" types, interacts with race/ethnicity) | Need (specific symptoms, severity) - interacts with predisposing (ethnicity) | | | 13,424 with lifetime depressed mood | 81.20% | | Strategy appropriate but no justification of sample size | | Yes (except Native Americans) | | Yes (though lifetime recall questionable) | | Yes | | ** | |
| Lopes et al. (2016) | | Brazil | | Cross-sectional | Adults (from nationally representative sample) | | Depression (PHQ-9) | | Current use of health services for depression | Gender (female), race/ethnicity (white), age (30-69), region (not the North), education (higher), multi-morbidities (includes both physical and mental). Null: marital status, urban/rural residence | Predisposing (gender, ethnicity, age, null: marital status), enabling (education), need (comorbid MH and other), contextual (region, null: urban/rural) | | | 4756 with depression | 86.10% | | Strategy appropriate but no justification of sample size | | Screener only | | No | | Yes | | ** | |
| Mackenzie et al. (2012) | | USA | | Cross-sectional | Community-dwelling adults (from nationally representative survey) | | Past year anxiety and mood disorders (AUDADIS-IV - DSM-IV criteria). Mood disorders includes mania, but disorders reported separately | | Past year contact with a health professional for mood/anxiety disorders (includes inpatient and emergency services) | Disorder type, age (interacted slightly with disorder type), gender (interacted slightly with age), comorbid anxiety/mood disorders (interacted slightly with age) | Predisposing (age, gender), need (comorbid anxiety/mood disorders, disorder type) | | | 9,487 with GAD, panic, phobia, social phobia, depression or dysthymia | 86.7% (for wave 2), 70.2% (from original sample - reported elsewhere) | | Strategy appropriate but no justification of sample size | | Yes | | Yes | | Yes | | **** | |
| Mojtabai & Olfson (2006) | | USA/Canada | | Cross-sectional | Adults with 12 month probable major depressive episode (from nationally representative sample) | | 12 month probable major depressive episode (CIDI-SF) | | 12 month contact with with a health professional for mental health reasons | US/Canadian residency made no difference to use/no use (but it did to source of care), race/ethnicity was associated in both countries, severity had more association in Canada than US | Predisposing (ethnicity) enabling (null: country of residence), need (severity) | | | 751 (304 from Canada and 447 from USA) | 66% (Canada), 50% (USA) | | Strategy appropriate but no justification of sample size (though see Sanmartin et al., 2004) | | Yes (representative of households with telephones) | | Screener only | | No | | ** | |
| Nakash et al. (2014) | | International | | Cross-sectional | Community-dwelling adults (from nationally representative surveys) | | CMD (CIDI) | | 12 month use of services for MH problems (either a MH professional or general medical professional) | Cancer status (active cancer, cancer survivor, cancer-free) | Need (comorbid disorders) | | | 14017 (active cancer; 96, cancer survivors; 355, cancer-free; 13566) | 79.9% (weighted - rates varied across countries) | | Strategy appropriate but no justification of sample size (though see Kessler & Ustun, 2008) | | Yes | | Yes | | Yes on average - in some countries no (e.g. France, 45.9%) | | *** | |
| Ojeda & McGuire (2006) | | USA | | Cross-sectional | Adults with depression/dysthymia (from nationally representative sample) | | Major depression/dysthymia (CIDI) | | 12 month use of outpatient mental health or substance use services (includes emergency services) | Education, age (interacts with gender), gender, perceived health status (interacts with gender), healthcare environment (i.e. managed care with gatekeeper), race/ethnicity, - interacts with gender (minority women less likely to use services than white women, African American men less likely to use services than white men but Latino men at same rate as white men). Ethnicity still associated after adjusting for education, insurance, and health status, (and age for AAs, young Latinos less likely to use services). Mental health status (severity?) and physical comorbidity not associated | Predisposing (gender, ethnicity, age), enabling (education, health care environment), need (perceived health status, null: severity, comorbidity) | | | 1498 | 64.0% (though non-responders includes those who were ineligible because they were children) | | Strategy appropriate but no justification of sample size | | Yes | | Yes | | Yes | | **** | |
| Olfson & Klerman (1992) | | USA | | Cross-sectional | Adults | | Depressive symptoms (DIS - DSM-III criteria) | | 6 month use of health services for MH reasons | Meeting diagnostic criteria associated with service use. Also age between 45 and 64 years, white, racial background, current unemployment, and separated or divorced marital status. Null: household income, education and gender (trends in expected directions but didn't reach significance) | Predisposing (age, ethnicity, marital status, null: gender), enabling (employment, null: income, education), need (meeting diagnostic criteria) | | | 744 with depressive symptoms | 68%-79% | | Strategy appropriate but no justification of sample size | | Yes (representative of communities sampled) | | Yes | | Yes | | **** | |
| Pirkis et al. (2001) | | Australia | | Cross-sectional | General population (adults only, from nationally representative sample) | | Anxiety disorders (CIDI) (affective disorders and SUDs also assessed, but reported separately - former included bipolar) | | Use of health services for mental health reasons (timeframe not specified, includes inpatient care) | English-speaking backgrounds (note: not ability to speak English, as non-English-speakers excluded) not associated with service use | Predisposing/enabling (linguistic background) | | | 10,641 (overall), 1026 with anxiety disorders | 78% | | Strategy appropriate but no justification of sample size | | Yes | | Timeframe of outcome not specified | | Yes | | *** | |
| Rafful et al. (2012) | | Mexico | | Cross-sectional | Urban community-dwelling residents aged 18 to 65 | | 12 month MDE (CIDI) | | 12 month consultation with a medic or other professional for emotional reasons | Gender (males more likely to seek help), interacted with age - youngest women least likely to seek help | Predisposing (gender - males more, interacts with age) | | | 531 with MDE | 76.60% | | Strategy appropriate but no justification of sample size | | Yes (representative of urban residents aged 18-65) | | Yes | | Yes | | *** | |
| Robinson et al. (2009) | | USA | | Cross-sectional | Individuals meeting criteria for an anxiety disorder in the past 12 months (from nationally representative sample) | | 12 month anxiety disorders (DSM-IV) | | Lifetime mental health service use (includes inpatient and emergency services) | Engaging in self-medication | Predisposing? (Doesn't obviously fit into any category) | | | 4880 | 81% | | Strategy appropriate but no justification of sample size | | Yes | | Yes (though lifetime recall questionable) | | Yes | | *** | |
| Rost et al. (1998) | | USA | | Prospective cohort | Adults who screen positive for depression on the DIS, from telephone survey | | "Substantial depressive symptoms" (screen-positive on DIS) | | 12 month treatment-seeking from a health professional for depression (self-report verified by medical/insurance records or recorded diagnosis/mention of depression in medical notes/antidepressant prescription). Inpatient treatment also measured but analysed separately | No rural-urban differences in service use (though affected number of specialty care visits). Gender, age, severity and psychiatric and physical comorbidity were associated with service use | Predisposing (gender, age), need (comorbidity, severity), enabling (null - neighbourhood level: urban/rural) | | | 446 | 74% (of whom 95% were followed up) | | Strategy appropriate but no justification of sample size | | Yes (representative of households with telephones) | | Screener only | | Yes | | *** | |
| Roy-Byrne et al. (2009) | | USA | | Cross-sectional | Adults who met criteria for a mood or anxiety disorder (from nationally representative sample) | | 12 month mood or anxiety disorder (CIDI) | | 12 month receipt of treatment for emotional or substance use problems (in general medical and mental health specialty sectors) | Age, gender, marital status, race/ethnicity associated, but education, income, and assets only minimally associated in multivariate analyses | Predisposing (age, gender, marital status, ethnicity), enabling (income, null: education, assets, urban-rural) | | | 1772 | 70.90% | | Strategy appropriate but no justification of sample size | | Yes | | Yes | | Yes | | **** | |
| Schomerus et al. (2013) | | Germany | | Prospective cohort | Adults aged 20–79 with lifetime depression (from population sample) | | Lifetime depression (M-CIDI) | | Lifetime visit to a psychologist, psychiatrist or general practitioner for their depressive symptoms | Age, education, perceived social support, childhood abuse, conscientiousness, resilience, depression severity. Gender, extraversion, openness, agreeableness, neuroticism, and alexithymia not associated | Predisposing (age, some personality factors), enabling (education, social support), need (childhood abuse*, severity). Predisposing (null - gender, other personality factors), need (null - alexithymia**). *alternatively could be seen as predisposing factor. ** alternatively could be seen as enabling factor | | | 354 | 68.8% (of whom 95.2% were invited to participate in the cohort study, of whom 65.4% agreed, of whom 5.6% were excluded due to missing data) | | Strategy appropriate but no justification of sample size | | Yes (representative of adults aged 20-79) | | Yes (though lifetime recall questionable) | | No | | ** | |
| Seedat et al. (2009) | | South Africa | | Cross-sectional | Adults living in households or hostels (from nationally representative sample) | | All disorders (CIDI 3.0) (separated for some analyses) | | 12 month contact with any mental health professional or general medical practitioner for MH (or substance use) reasons | Gender (p=0.05 for depression/dysthymia, p=0.77 for anxiety disorders). No socio-demographic variables associated: age, education, race, marital status, income. (There were some associations found within gender groups but these combined those with CMD and SUD) | Predisposing (gender, but only for depression not anxiety, null: age, ethnicity, marital status), enabling (null: education, income) | | | 4351 | 85.50% | | Strategy appropriate but no justification of sample size | | Yes | | Yes | | Yes | | **** | |
| Starkes et al. (2005) | | Canada | | Cross-sectional | General population (aged 12+) from population survey | | Major depression (CIDI-SF) | | 12 month consultation with a health professional about mental or emotional health | Age (several other variables investigated but included those under age 18) - older age associated with reduced chances of help-seeking, though only 45-64 year old group significantly lower, 65+ not statistically different from 20-44 year olds | Predisposing (age) | | | 1,312 | 80% (not reported in article but found elsewhere) | | Sampling strategy not specified. Sample size calculated to have sufficient (unspecified) power to estimate the numbers seeking treatment | | Yes | | Yes | | Yes | | *** | |
| Sussman et al. (1987) | | USA | | Cross-sectional | General population (presumably adults only), from urban household survey | | Depression (DSM-III criteria using DIS) (episode in past 6 months) | | Two measures; (1) 6 month disclosure of emotional/psychological problems as an outpatient/1 year disclosure as an inpatient (includes to non-health providers), (2) lifetime disclosure of depressive symptoms to a doctor or (non-specified) professional | Race/ethnicity, which interacts with severity (racial disparities greatest in those with mild symptoms) | Predisposing (ethnic group) and need (severity) | | | 116 | 75%-80%, depending on the site (not reported in article but found elsewhere) | | Strategy appropriate but no justification of sample size | | Yes (when weighted). Includes those in institutions | | Yes | | Yes | | **** | |
| Tempier et al. (2009) | | Australia/Canada | | Cross-sectional | General adult population (excludes under-18s from Canadian sample) | | 12 month depressive/anxiety disorders (CIDI) (also measures SUD but presented separately) | | 12 month use of outpatient health services for MH reasons | Country associated for comorbid anxiety/depression but not depression alone - borderline for anxiety alone (p=0.05) | Contextual enabling (country), interacts with need (disorder type) | | | Not specified. Total original sample size was 36816 (Canada) and 10641 (Australia) but this includes under-18s in Canada | 78% (Australia), 77% (Canada) | | Strategy appropriate but no justification of sample size | | Yes | | Yes | | Yes | | **** | |
| Tempier et al. (2010) | | Canada/France/Belgium | | Cross-sectional | General adult population | | 12 month depressive/anxiety disorders (CIDI) | | 12 month contact with a health professional for MH reasons (lifetime also measured) | Overall, no statistically significant differences found for 12 month service use (although the French use psychiatrists more and Canadians use non-psychiatrist MH professionals more). Once separated by disorder, Canadians with anxiety disorders used services more than Europeans but no stat. sig. difference for MDE or combination | Contextual enabling (country), interacts with need (disorder type) | | | Number with disorders not specified. Total original sample size was 8071 (Canada), 389 (Belgium), 1436 (France) | 77% (Canada), 51% (Belgium), 46% (France) | | Strategy appropriate but no justification of sample size | | Yes | | Yes | | No (only in Canada) | | *** | |
| ten Have et al. (2004) | | Netherlands | | Prospective cohort | General population aged 18–64 | | Lifetime major or minor depression (CIDI - DSM-III-R criteria) | | Lifetime use of health services for MH reasons | Associated with both primary and specialist care: Fatigue/loss of energy, feelings of worthlessness or guilt, comorbid anxiety disorder, having a parent with a psychiatric history. Associated with specialist care only: loss of interest/pleasure, weight loss/gain, insomnia/hypersomnia, reduced ability to think/concentrate, age, education, age of onset (reduces chances of treatment, whilse all other increase it). Associated with primary care only: comorbid SUD. Null: depressed mood, psychomotor agitation/retardation, recurrent thoughts of death, gender. | Predisposing (age and age of onset associated with specialist care only (latter negatively), null: gender), enabling (education associated with specialist care only), need (specific symptoms: fatigue, feelings of guilt/worthlessness, comorbid anxiety, parent with history of MH problems, SUD associated with primary care only, various symptoms associated with specialist care only, null: depressed mood, psychomotor agitation/retardation, recurrent thoughts of death) | | | 1572 with lifetime major or minor depression | 69.7%, 79.4 % followed up | | Strategy appropriate but no justification of sample size | | Yes (representative of 18-64 year-olds) | | Yes (though lifetime recall questionable) | | Yes | | ** | |
| Vasiliadis et al. (2007) | | USA/Canada | | Cross-sectional | Community-dwelling adults (aged 18+) | | Probable MDE (CIDI-SF - DSM-III-R criteria) | | 12 month contact with a health professional for MH reasons | Health insurance (interacts with country - no effect in Canada, only in USA, though this disappeared in fully adjusted models). Disability (borderline significant? No p-value presented, 95% CI for OR: 1.0-1.7), long-term health problems that affect daily life, having a regular medical doctor, gender (female), marital status (single), education (university vs. less than high school), race (white vs. other). Null overall: income (but affects use of specialist services), perceived general health (affects use of generalist services), age, country of birth, household size. (Unmet need not associated with overall use/no use) | Predisposing (gender, marital status, ethnicity, null: age, country of birth) enabling (insurance - interacts with country, education, null: income - but affects use of specialist care, household size), need (disability borderline, chronic conditions that affect daily life, null: perceived general health - but affects use of generalist services) | | | 287 (Canada), 451 (USA) with MDE | 66% (Canada), 50% (USA) | | Strategy appropriate but no justification of sample size | | Yes | | Screener only | | No (only in Canada) | | ** | |
| Vesga-Lopez et al. (2008) | | USA | | Cross-sectional | General population aged 18+ (from nationally representative sample) | | Generalised anxiety disorder (DSM-IV criteria) | | Lifetime contact with a counselor, therapist, physician, or psychologist, inpatient admission, use of emergency services, or receipt of prescription for psychotropic medication | Gender | Predisposing (gender) | | | 1757 | 81% | | Strategy appropriate but no justification of sample size | | Yes | | Yes | | Yes | | **** | |
| Vigod & Levitt (2011) | | Canada | | Cross-sectional | General population aged 20+ (from telephone survey) | | Lifetime depressive symptoms (CIDI) | | Lifetime depression-specific use of health services from a physician (family physician or psychiatrist) - also looked at Lifetime psychotropic medication use, use of health services from a non-physician therapist, and psychiatric hospitalization | Seasonal depression severity score | Need (seasonality - positive association) | | | 625 | 81.10% | | Not 100% clear - area stratified by latitude and then presumably sampled using PPS methods, but not specified. No discussion of sample size. | | Mostly (sample not significantly different from the Ontario population with regard to educational level, marital status, and employment status, but women slightly oversampled and not clear whether rural-urban divide was representative) | | Yes (though lifetime recall questionable) | | Yes | | ** | |
| Wallerblad et al. (2012) | | Sweden | | Prospective cohort | Swedish citizens aged 20–64 years from one county | | Depressive/anxiety disorders (multiple diagnostic tools - DSM-IV criteria) | | 12 month contact with a health professional for psychological/sleeping/personal problems (includes "alternative medical treatment" - undefined) | Gender (female), age (older), marital status (single), nativity (born abroad), employment (outside the labour market), comorbid somatic illness, comorbid mental illness (both depression and anxiety), severity, disability | Predisposing (gender, age, marital status, nativity), enabling (employment), need (comorbid somatic illness, comorbid mental illness, severity, disability) | | | 2026 with CMD | 53%, retention rate 83% (so overall 44%) | | Strategy appropriate but no justification of sample size | | Yes (representative of 20-64 year-old citizens from region surveyed) | | Yes | | No | | ** | |
| Wang et al. (2000) | | USA | | Cross-sectional | Adults (from nationally representative sample) | | MDD, panic disorder or GAD (CIDI-SF) | | 12 month use of health care for MH reasons, or self-help or religious advisor | Education level and number of comorbid conditions predicted use of generalist sector, while severity, age and MH insurance predicted use of specialist sector. Severity, comorbidity and insurance coverage predicted any treatment-seeking (including from non-health sector) | Need (disorder type) | | | 428 (depression), 206 (panic disorder), 100 (GAD) (558 in total, presumably due to overlap in categories with comorbidities) | 60.8% (70% for initial sampling stage and 86.6% for full interview) | | Strategy appropriate but no justification of sample size | | Yes (when weighted) | | No outcome that corresponds to any health service use | | Borderline | | ** | |
