# Supplementary material for: Factors associated with health service utilisation for common mental disorders: a systematic review
Source: BMC Psychiatry. 2018 Aug 22;18:262. doi: 10.1186/s12888-018-1837-1 (PMC6104009; doi:10.1186/s12888-018-1837-1)
Supplement: Supplementary file 4 — Detailed results by factor. (DOCX 141 kb) [file 12888_2018_1837_MOESM4_ESM.docx]

**Additional file 4 Detailed results by factor**

To avoid repetition in the presentation of results, when a factor is said to have been investigated, this should be understood as “investigated as a potential correlate of health service utilisation for CMD”. Any associations referred to should be understood as associations between this factor and health service utilisation for CMD, among individuals who screen positive for CMD.

#### Predisposing factors

##### Summary

As shown in table 5, while there were some trends towards greater treatment-seeking by women, the middle-aged and individuals in the majority ethnic group, and having sought treatment for a previous episode of CMD symptoms, none of these associations was identified consistently across studies.

##### Age

Twenty-five studies investigated age. Fifteen studies were of good/excellent quality, and 18 of 25 studies reported some association. The most common relationship found was hill-shaped, with middle-aged respondents most likely to seek treatment (1-12). Three studies reported lower use by the youngest or oldest age groups (13-15), one study found a positive association with age (16), two studies reported mixed findings (17, 18) and seven studies found no evidence of a correlation (19-25).

##### Age of onset

Three studies tested age of onset of CMD, of which two were rated good/excellent quality. In two out of three papers a later onset was associated with increased likelihood of seeking treatment (26, 27), while Ten Have et al. (2004) reported mixed findings (18).

##### Gender

Twenty-nine studies investigated either gender or sex, of which eighteen were rated good/excellent quality, and 20 of 29 reported some association. With the exception of Rafful et al. (2012) (17), all of the studies that reported gender differences found that women were more likely to seek help than men (1, 2, 6, 7, 9, 11, 14, 25, 26, 28-30). Six studies reported mixed results (4, 5, 13, 21, 27, 31), while nine studies reported no evidence of an association (3, 10, 12, 15, 16, 18, 19, 22, 24).

##### Ethnicity

Twenty-three studies investigated race or ethnicity. Seventeen were of good/excellent quality, and 20 of 23 reported some association. It was commonly reported that Causasians (the majority ethnic group in the context of most studies) were more likely to seek treatment than minority ethnic groups (1, 7, 9-11, 25, 28, 32-36). Some studies found differences between minority groups, but these were not consistent with one another (12, 37, 38). Six studies reported mixed results (5, 21, 27, 39-41) and three did not find any association (2, 3, 30).

##### Education

Twenty studies examined education level. Thirteen were rated good/excellent quality and 11 of 20 reported some association. All those that found an association reported that higher education levels were associated with a greater likelihood of seeking treatment (1, 5, 9, 11, 16, 19, 25, 27, 28). Two reported mixed results (18, 20) while nine found no evidence of an association (2, 3, 7, 10, 12, 15, 21, 22, 30).

##### Immigration status/country of birth

Six studies investigated country of origin or linguistic background. Two were classified as good/excellent quality and 3 of 6 reported some association. Three studies found no evidence of any association (25, 27, 42), one reported mixed results (36) and two studies found associations in opposite directions (14, 43).

##### Marital status

Eighteen studies investigated marital status. Fourteen were rated as good/excellent quality, and 10 of 18 reported some association. In general, being married was associated with a lower likelihood of treatment-seeking, though it is unclear from the evidence whether this was due to greater use of services by the separated or divorced group, or the never married (3, 7, 10, 11, 14, 25, 30). One study found that married individuals were more likely to seek treatment than other groups (28). Eight studies reported no evidence of an association (1, 2, 9, 19, 21-24) and one found mixed results (27).

Three studies examined a change in marital status. The findings were inconsistent (26, 27, 30).

##### Personality factors

Four studies examined personality traits. Two were of good/excellent quality. 3 of 4 reported some association. There was no clear consensus between studies as to which traits were associated with seeking treatment (3, 16, 23, 31).

##### Attitudes towards and experience of mental health services

Five studies reported on previous experiences with mental health services or expectations of mental health services. Two were rated good/excellent quality. 2 of 3 reported a positive association with having prior experience of services (27, 30, 43), one found a positive association with expected benefits of services (2), and one very small study did not find an association with trust in professional services (24).

##### Stigma

Two studies, of which one was rated good quality, examined stigma. One found an association with personal stigma but not perceived stigma (23), while the other – a very small study – found no evidence of an association (24).

#### Enabling factors (individual level)

##### Summary

As indicated in table 5, there was inconsistent evidence for an association between treatment-seeking for CMD and enabling factors. The studies included here suggest that income is not associated with the use of health services for CMD symptoms.

##### Income/wealth

Eleven studies assessed wealth or income. Eight were of good/excellent quality. Just one of 11 studies found a positive association with income (the measure most commonly used) (1), two found mixed results (7, 25) and a fourth reported a negative association with concerns about affordability (24). None of the remaining studies found an association (10, 12, 13, 19, 21, 22, 30).

##### Employment

Eight studies examined employment. Four were classified as good/excellent quality. 4 of 8 studies found a negative association with being in employment (2, 10, 14, 22), while the others did not find an association (3, 13, 15, 19).

##### Social support

Five studies examined social support or related factors, of which one was rated as good/excellent quality. Two of five studies reported an association, with greater perceived social support linked to treatment seeking (13, 16), and two did not (2, 23). No association was found with household size (25).

##### Insurance

Seven studies reported on health insurance. Three were of good/excellent quality. Four studies reported a positive association between having health insurance and treatment-seeking (1, 2, 5, 12). One reported mixed results (20) and two did not find an association (25, 30).

##### Regular source of care

Two studies – of which one was rated good/excellent – investigated having a usual source of health care, with inconsistent results (30, 43).

#### Need factors (individual level)

##### Summary

Need factors were most consistently associated with the use of health services for CMD symptoms across studies, as seen in table 5. Chronicity or duration of symptoms, disability (particularly affecting the ability to work), comorbid mental disorders, panic symptoms, and self-rated health status or perceived need for care, were all associated with treatment-seeking, and there was a trend towards greater treatment-seeking by those with more severe symptoms.

##### Self-rated health / Perceived need for care

Eight studies examined individuals’ perceptions of their own health status or need for care. Four were rated as good/excellent quality. 3 of 8 studies reported a negative association between treatment-seeking and self-rated health status or subjective distress (5, 28, 31). Two studies found indirect evidence for the role of perceived need for care in mediating or moderating the effects of ethnicity on treatment-seeking (37, 40). Two studies did not find an association (2, 24), including one longitudinal study, while another reported mixed results (25). Two out of three longitudinal studies reported a negative association.

##### Symptom severity

Sixteen studies investigated symptom severity. Eight were of good/excellent quality. 10 of 16 studies found a positive correlation between symptom severity and treatment-seeking (2, 3, 6, 10, 11, 14, 16, 32, 33, 39) and another found mixed results (15). Five studies did not report an association (5, 22, 24, 26, 31). Five out of six longitudinal studies found a positive association.

##### Chronicity/Duration

Three studies examined the chronicity or duration of illness, all of which were rated as good/excellent quality (1, 23, 26). All found a positive association. None used longitudinal data.

##### Disability

Eight studies investigated some measure of disability or functioning. Three were rated good/excellent quality, and 7 of 8 reported some association. Five studies reported that those with greater levels of impairment were more likely to seek treatment (3, 14, 15, 22, 43), two found mixed results (20, 31) and the final study found an association that bordered on statistical significance (25). Of two longitudinal studies, one reported a positive association.

##### Comorbid conditions - total

Four studies reported on an individual’s total number of comorbid conditions, of which one was rated as good/excellent quality. 3 of 4 studies – including the only longitudinal study – reported no evidence of an association (9, 26, 31) while Wang et al. (2000) reported mixed results (20).

##### Non-psychiatric chronic conditions

Fourteen studies investigated non-psychiatric comorbidities. Ten were of good/excellent quality. Only three of the eight studies that looked at medical comorbidities in general found a positive association (6, 14, 25), while five reported no evidence of an association (2, 3, 5, 22, 23). Two out of three longitudinal studies reported a positive association. Bucholz and Robins (1987) found an association with worsening physical health (30).

Five studies investigated specific comorbid conditions or comorbid pain, with mixed results (1, 13, 44-46).

##### Psychiatric comorbidity

Seventeen studies investigated comorbid mental and substance use disorders. Fourteen were classified as of good/excellent quality.

Of the six studies that examined comorbid mental disorders in general, all – including all three longitudinal studies – reported a positive association with treatment-seeking, (2, 3, 6, 11, 15, 22).

Six studies investigated comorbid mood or anxiety disorders. Five reported a positive association (4, 14, 18, 27, 28) while one did not (23). All four longitudinal studies found a positive association.

Eight studies examined substance use symptoms. Three found a positive association (28, 47, 48), two reported a negative association (26, 49), one reported mixed results (18) and two did not find an association (1, 27). The findings from longitudinal studies were equally mixed.

Findings with regard to other comorbid psychiatric diagnoses were inconsistent (1, 26, 49).

##### Specific CMD symptoms

Thirteen studies investigated specific symptom profiles. Ten were of good/excellent quality.

Five studies reported that panic symptoms were associated with treatment-seeking (3, 4, 26, 27, 49) although one found reduced treatment-seeking by those with panic symptoms compared to those without (20). There were two longitudinal studies of which both reported a positive association.

Three studies investigated suicidality, with inconsistent results (11, 15, 26). There was one longitudinal study, which did not find an association.

There was no clear consensus on the associations between other CMD symptoms and treatment-seeking (18, 23, 30, 39, 50) and no evidence of an association with somatisation (26, 49).

##### Adverse childhood events

Four studies examined adverse life events in childhood of various sorts, of which one was rated as good/high quality. Three found that adults who had experienced adversity as a child were more likely to seek treatment (16, 18) while two reported mixed results (15, 31).

As mentioned under “predisposing factors”, there were contradictory findings with regard to bereavement or change in marital status (26, 27, 30).

#### Contextual level factors

##### Summary

Limited evidence was found for the association between contextual level factors and health service utilisation for CMD. The studies included here suggest that living in a rural area is not associated with lower rates of treatment-seeking.

##### Place of residence

###### Urban/rural

Seven studies examined urban or rural residence, of which 6 of 7 were classified as good/excellent quality. None found evidence of an association overall (1, 3, 6, 7, 9, 15, 22).

###### Country

Three studies, of which two were of good/excellent quality, compared treatment-seeking by country of residence. There were no consistent findings, despite the differences in health systems between the countries included (32, 51, 52).

###### Within-country region

Three studies examined differences by within-country region. 2 of 3 were rated as good/excellent quality. 2 reported an association (9, 11), while one did not find an association (1).

##### Health care environment

###### Organisation of services

One study, rated excellent quality, examined the effect of managed care and found that that those for whom there is a gatekeeper to health services are less likely to seek treatment (5).

###### Availability of services

Only one study, rated “fair” quality, examined perceived availability of services, and found a positive association with treatment-seeking (2).

###### Accessibility of services

One study, of poor quality and a very small sample size, investigated concerns about the accessibility of health services (24). No evidence of an association was found.

## References

1. Carragher N, Adamson G, Bunting B, McCann S. Treatment-seeking behaviours for depression in the general population: results from the National Epidemiologic Survey on Alcohol and Related Conditions. Journal of affective disorders. 2010;121(1):59-67.

2. Fortney J, Rost K, Zhang M. A joint choice model of the decision to seek depression treatment and choice of provider sector. Medical Care. 1998;36(3):307-20.

3. Issakidis C, Andrews G. Service utilisation for anxiety in an Australian community sample. Social psychiatry and psychiatric epidemiology. 2002;37(4):153-63.

4. Mackenzie CS, Reynolds K, Cairney J, Streiner DL, Sareen J. Disorder‐specific mental health service use for mood and anxiety disorders: Associations with age, sex, and psychiatric comorbidity. Depression and anxiety. 2012;29(3):234-42.

5. Ojeda VD, McGuire TG. Gender and racial/ethnic differences in use of outpatient mental health and substance use services by depressed adults. Psychiatric Quarterly. 2006;77(3):211-22.

6. Rost K, Zhang M, Fortney J, Smith J, Smith Jr GR. Rural-urban differences in depression treatment and suicidality. Medical Care. 1998;36(7):1098-107.

7. Roy-Byrne PP, Joesch JM, Wang PS, Kessler RC. Low socioeconomic status and mental health care use among respondents with anxiety and depression in the NCS-R. Psychiatric Services. 2009;60(9):1190-7.

8. Starkes JM, Poulin CC, Kisely SR. Unmet need for the treatment of depression in Atlantic Canada. The Canadian Journal of Psychiatry. 2005;50(10):580-90.

9. Lopes CS, Hellwig N, e Silva GdA, Menezes PR. Inequities in access to depression treatment: results of the Brazilian National Health Survey–PNS. International journal for equity in health. 2016;15(1):154.

10. Olfson M, Klerman G. Depressive symptoms and mental health service utilization in a community sample. Social Psychiatry and Psychiatric Epidemiology. 1992;27(4):161-7.

11. Chartrand H, Robinson J, Bolton JM. A longitudinal population-based study exploring treatment utilization and suicidal ideation and behavior in major depressive disorder. Journal of affective disorders. 2012;141(2):237-45.

12. González HM, Vega WA, Williams DR, Tarraf W, West BT, Neighbors HW. Depression care in the United States: too little for too few. Archives of general psychiatry. 2010;67(1):37-46.

13. Andersson LM, Schierenbeck I, Strumpher J, Krantz G, Topper K, Backman G, et al. Help-seeking behaviour, barriers to care and experiences of care among persons with depression in Eastern Cape, South Africa. Journal of affective disorders. 2013;151(2):439-48.

14. Wallerblad A, Möller J, Forsell Y. Care-seeking pattern among persons with depression and anxiety: a population-based study in Sweden. International journal of family medicine. 2012;2012.

15. Hamalainen J, Isometsa E, Sihvo S, Pirkola S, Kiviruusu O. Use of health services for major depressive and anxiety disorders in Finland. Depression and anxiety. 2008;25(1):27-37.

16. Schomerus G, Appel K, Meffert PJ, Luppa M, Andersen RM, Grabe HJ, et al. Personality-related factors as predictors of help-seeking for depression: a population-based study applying the behavioral model of health services use. Social psychiatry and psychiatric epidemiology. 2013;48(11):1809-17.

17. Rafful C, Medina-Mora ME, Borges G, Benjet C, Orozco R. Depression, gender, and the treatment gap in Mexico. Journal of affective disorders. 2012;138(1):165-9.

18. ten Have M, de Graaf R, Vollebergh W, Beekman A. What depressive symptoms are associated with the use of care services?: results from the Netherlands Mental Health Survey and Incidence Study (NEMESIS). Journal of affective disorders. 2004;80(2):239-48.

19. Hailemariam S, Tessema F, Asefa M, Tadesse H, Tenkolu G. The prevalence of depression and associated factors in Ethiopia: findings from the National Health Survey. International journal of mental health systems. 2012;6(1):23.

20. Wang PS, Berglund P, Kessler RC. Recent care of common mental disorders in the United States. Journal of general internal medicine. 2000;15(5):284-92.

21. Seedat S, Williams DR, Herman AA, Moomal H, Williams SL, Jackson PB, et al. Mental health service use among South Africans for mood, anxiety and substance use disorders. South African Medical Journal. 2009;99(5).

22. Gabilondo A, Rojas-Farreras S, Rodráguez A, Ferníndez A, Pinto-Meza A, Vilagut G, et al. Use of primary and specialized mental health care for a major depressive episode in Spain by ESEMeD respondents. Psychiatric Services. 2011;62(2):152-61.

23. Boerema AM, Kleiboer A, Beekman AT, van Zoonen K, Dijkshoorn H, Cuijpers P. Determinants of help-seeking behavior in depression: a cross-sectional study. BMC psychiatry. 2016;16(1):78.

24. Chen J. Seeking help for psychological distress in urban China. Journal of Community Psychology. 2012;40(3):319-41.

25. Vasiliadis H-M, Lesage A, Adair C, Wang PS, Kessler RC. Do Canada and the United States differ in prevalence of depression and utilization of services? Psychiatric Services. 2007;58(1):63-71.

26. Galbaud du Fort G, Newman S, Boothroyd L, Bland R. Treatment seeking for depression: role of depressive symptoms and comorbid psychiatric diagnoses. Journal of affective disorders. 1999;52(1):31-40.

27. Iza M, Olfson M, Vermes D, Hoffer M, Wang S, Blanco C. Probability and predictors of first treatment contact for anxiety disorders in the United States: analysis of data from the National Epidemiologic Survey on Alcohol and Related Conditions (NESARC). The Journal of clinical psychiatry. 2013;74(11):1093-100.

28. Burnett-Zeigler I, Zivin K, Islam K, Ilgen MA. Longitudinal predictors of first time depression treatment utilization among adults with depressive disorders. Social psychiatry and psychiatric epidemiology. 2012;47(10):1617-25.

29. Vesga-López O, Schneier F, Wang S, Heimberg R, Liu S-M, Hasin DS, et al. Gender differences in generalized anxiety disorder: results from the National Epidemiologic Survey on Alcohol and Related Conditions (NESARC). The Journal of clinical psychiatry. 2008;69(10):1606.

30. Bucholz KK, Robins LN. Who talks to a doctor about existing depressive illness? Journal of affective disorders. 1987;12(3):241-50.

31. Angst J, Gamma A, Clarke D, Ajdacic‐Gross V, Rössler W, Regier D. Subjective distress predicts treatment seeking for depression, bipolar, anxiety, panic, neurasthenia and insomnia severity spectra. Acta Psychiatrica Scandinavica. 2010;122(6):488-98.

32. Mojtabai R, Olfson M. Treatment seeking for depression in Canada and the United States. Psychiatric Services. 2006;57(5):631-9.

33. Sussman LK, Robins LN, Earls F. Treatment-seeking for depression by black and white Americans. Social Science & Medicine. 1987;24(3):187-96.

34. Alegría M, Chatterji P, Wells K, Cao Z, Chen C-n, Takeuchi D, et al. Disparity in depression treatment among racial and ethnic minority populations in the United States. Psychiatric services. 2008;59(11):1264-72.

35. Keyes KM, Hatzenbuehler ML, Alberti P, Narrow WE, Grant BF, Hasin DS. Service utilization differences for Axis I psychiatric and substance use disorders between white and black adults. Psychiatric Services. 2008;59(8):893-901.

36. Bauldry S, Szaflarski M. Immigrant-based Disparities in Mental Health Care Utilization. Socius. 2017;3:2378023116685718.

37. Ault-Brutus AA. Changes in racial-ethnic disparities in use and adequacy of mental health care in the United States, 1990–2003. Psychiatric Services. 2012;63(6):531-40.

38. Lee SY, Martins SS, Keyes KM, Lee HB. Mental health service use by persons of Asian ancestry with DSM-IV mental disorders in the United States. Psychiatric Services. 2011;62(10):1180-6.

39. Lee SY, Xue Q-l, Spira AP, Lee HB. Racial and ethnic differences in depressive subtypes and access to mental health care in the United States. Journal of affective disorders. 2014;155:130-7.

40. Ault-Brutus A, Alegria M. Racial/ethnic differences in perceived need for mental health care and disparities in use of care among those with perceived need in 1990–1992 and 2001–2003. Ethnicity & health. 2016:1-16.

41. Hankerson SH, Fenton MC, Geier TJ, Keyes KM, Weissman MM, Hasin DS. Racial differences in symptoms, comorbidity, and treatment for major depressive disorder among black and white adults. Journal of the National Medical Association. 2011;103(7):576-84.

42. Pirkis J, Burgess P, Meadows G, Dunt D. Access to Australian mental health care by people from non‐English‐speaking backgrounds. Australian and New Zealand Journal of Psychiatry. 2001;35(2):174-82.

43. Gwynn RC, McQuistion HL, McVeigh KH, Garg RK, Frieden TR, Thorpe LE. Prevalence, diagnosis, and treatment of depression and generalized anxiety disorder in a diverse urban community. Psychiatric Services. 2008;59(6):641-7.

44. Nakash O, Levav I, Aguilar-Gaxiola S, Alonso J, Andrade LH, Angermeyer MC, et al. Comorbidity of common mental disorders with cancer and their treatment gap: findings from the World Mental Health Surveys. Psycho-Oncology. 2014;23:40-51.

45. Demyttenaere K, Bonnewyn A, Bruffaerts R, Brugha T, De Graaf R, Alonso J. Comorbid painful physical symptoms and depression: prevalence, work loss, and help seeking. Journal of affective disorders. 2006;92(2):185-93.

46. Demyttenaere K, Bonnewyn A, Bruffaerts R, De Graaf R, Haro JM, Alonso J. Comorbid painful physical symptoms and anxiety: prevalence, work loss and help-seeking. Journal of affective disorders. 2008;109(3):264-72.

47. Chen L-Y, Crum RM, Martins SS, Kaufmann CN, Strain EC, Mojtabai R. Service use and barriers to mental health care among adults with major depression and comorbid substance dependence. Psychiatric services. 2013;64(9):863-70.

48. Robinson JA, Sareen J, Cox BJ, Bolton JM. Correlates of self-medication for anxiety disorders: results from the National Epidemiolgic Survey on Alcohol and Related Conditions. The Journal of nervous and mental disease. 2009;3(12):873-8.

49. Bucholz KK, Dinwiddie SH. Influence of nondepressive psychiatric symptoms on whether patients tell a doctor about depression. The American journal of psychiatry. 1989;146(5):640.

50. Vigod SN, Levitt AJ. Seasonal severity of depressive symptoms as a predictor of health service use in a community-based sample. Journal of psychiatric research. 2011;45(5):612-8.

51. Tempier R, Meadows GN, Vasiliadis H-M, Mosier KE, Lesage A, Stiller A, et al. Mental disorders and mental health care in Canada and Australia: comparative epidemiological findings. Social psychiatry and psychiatric epidemiology. 2009;44(1):63-72.

52. Tempier R, Vasiliadis H-M, Gilbert F, Demyttenaere K, Bruffaerts R, Lépine J-P, et al. Comparing mental health of francophones in Canada, france, and belgium: 12-month and lifetime rates of mental health service use (part 2). The Canadian Journal of Psychiatry. 2010;55(5):295-304.
